# Pharmacist-led adherence support in general practice: a qualitative interview study of adults with asthma

**DOI:** 10.1136/bmjopen-2019-032084

**Published:** 2019-11-06

**Authors:** Marissa Ayano Mes, Caroline Brigitte Katzer, Vari Wileman, Amy Hai Yan Chan, Robert Horne, Stephanie Jane Caroline Taylor

**Affiliations:** 1 Centre for Behavioural Medicine, Research Department of Practice and Policy, UCL School of Pharmacy, London, UK; 2 Centre for Primary Care and Public Health, Blizard Institute, Queen Mary University of London, London, UK

**Keywords:** medication adherence, asthma, pharmaceutical services, general practice, medication therapy management

## Abstract

**Objectives:**

The National Health Service (NHS) in England recently introduced general practice pharmacists (GPPs) to provide medication-focused support to both patients and the general practice team. This healthcare model may benefit people with asthma, who currently receive suboptimal care and demonstrate low medication adherence. This study aimed to explore the perspectives of adults with asthma on the potential for pharmacist-led adherence support delivered in general practice, with a focus on how these perspectives are formed.

**Design and setting:**

The study was conducted in the United Kingdom (UK) utilising a qualitative interview methodology. Participants were invited to partake in a telephone-based semistructured interview, followed by an online questionnaire for demographic details and asthma history. Qualitative data were analysed using thematic analysis.

**Participants:**

Participants (n=17) were adults with asthma in the UK with a prescription for an inhaled corticosteroid. Participants did not have previous experience with GPPs and were asked to provide their views on a proposed GPP-led service.

**Results:**

Participant perspectives of GPPs were determined by trust in pharmacists, perceived gaps in asthma care and the perceived strain on the NHS. Trust was based on pharmacists’ perceived clinical competency, established over time, and gauged through a ‘benchmarking’ process. GPP’s fit in current asthma care was assessed based on potential role overlap with other healthcare professionals, continuity of care and medication-related support needs. Participants navigated the NHS based on a perceived hierarchy of healthcare professionals (general practitioners on top, nurses, then pharmacists), and this influenced their perspectives of GPPs.

**Conclusion:**

While the GPP scheme shows promise based on the perspectives of people with asthma, the identified barriers to optimal patient engagement and service implementation will need to be addressed for the service to be effective.

**Strengths and limitations of this study**

- The use of qualitative methodology captured the complex processes behind people’s perspectives of general practice pharmacists (GPPs), including how lived experiences shaped opinions of new pharmacist-led services.
- Telephone-based interviews enabled recruitment across the UK and increased study accessibility for people with severe asthma and travel limitations.
- The study may not have captured the full variation in views among adults with asthma in the UK because participants primarily had self-reported mild asthma and were recruited through an asthma charity.
- Participants had no experience with GPP-led consultations and therefore represented the general population with asthma that would initially need to be convinced to engage with the service.

## Background

The pressure on primary care to deliver core services is increasing rapidly worldwide due to a growing and ageing population, the prevalence of long-term conditions and significant resource constraints. Primary care systems are being reshaped, and new models of care are emerging to cope with growing demand.^1^

One such model is the general practice pharmacist (GPP) model, which was introduced in England as part of the NHS *General Practice Forward View* initiative.^2–4^ These pharmacists support both the patients and the general practice team with medication-related issues, with the aim of expanding the general practice workforce, reducing practice burden and increasing patient access to appointments.^4^ Initial qualitative feedback from general practitioners (GPs) and pharmacists in a pilot study in England suggests that GPPs can have positive impacts on medication safety, medication adherence, healthcare access and patient satisfaction across a variety of long-term conditions.^3^

People with asthma may benefit from GPP support. Research shows that adherence to inhaled corticosteroids (ICS), an essential medication for asthma, is low.^5 6^ Furthermore, only 35% of people with asthma in the UK receive basic care (ie, annual reviews, inhaler technique checks and a written asthma action plan).^7^ Previous research suggests that pharmacist-led interventions can increase medication adherence and support asthma self-management among people with asthma.^8^ However, most of the previous research in the UK has been limited to community rather than GPPs and focused on their impact across a range of long-term conditions rather than asthma specifically.^9 10^

Understanding the specific perspectives of people with asthma regarding GPPs is an important first step in establishing the potential benefits of this new service for this specific patient group, as well as identifying any potential issues in its future uptake and effectiveness.^11^ The aim of this study was to explore the perspectives of adults with asthma on the potential of pharmacist-led asthma adherence support delivered in general practice, with a focus on how these perspectives are formed.

## Methods

This study is reported according to the Standards for Reporting Qualitative Research.^12^ It was a telephone-based semistructured interview study, using an interpretivist approach to understand how adults with asthma construct their initial opinions of GPP-led asthma adherence support. Demographic details and asthma history were collected using a brief online questionnaire.

### Participants

Participants were adults (≥18 years old) living in the UK and proficient in English, with a self-reported asthma diagnosis, a prescription for ICS and access to a telephone and e-mail account. People with respiratory comorbidities (eg, Chronic Obstructive Pulmonary Disease) and/or those in hospital or nursing homes were excluded, as the adherence behaviour and support needs of these individuals were hypothesised to be different.

### Recruitment

Several recruitment channels were used to ensure that participants varied in age, gender and self-reported asthma severity. A flyer with study information and researchers’ contact details was circulated by researchers, the Asthma UK Centre for Applied Research (AUKCAR) and the National Institute for Health Research Collaboration for Leadership in Applied Health Research and Care North Thames via social media. The study was advertised in two electronic newsletters: the Asthma UK volunteer bulletin and the University College London student newsletter. Printed flyers were handed directly to potential participants by a respiratory consultant at a London hospital and pharmacists at two hospitals in Wales.

People who contacted the researchers were e-mailed an information sheet, consent form and eligibility criteria to review. If they were eligible and willing to participate, they were booked in for a 1-hour telephone interview. In preparation for the call, participants were asked to read a description of a GPP-led adherence support consultation, sent to them via e-mail (see online supplementary appendix A). This description was based on the work of a clinical respiratory pharmacist working in general practice in London. Participants gave verbal consent over the telephone before the interview began. The consent procedure and interview were audio-recorded with their permission. All participants received a £20 online shopping voucher to thank them for their time.

10.1136/bmjopen-2019-032084.supp1 Supplementary data

### Data collection and analysis

One researcher (MAM) conducted the interviews. Participants were informed that the researcher had a background in Health Psychology and an interest in adherence and pharmacist-led care for asthma.

The interview topic guide had two sections (see online supplementary appendix B). The first section focused on participants’ previous experiences of asthma, asthma care and pharmacists. The second section focused on how these lived experiences informed participants’ opinions of GPP-led adherence support, with questions based on previous research on interpersonal/institutional trust in healthcare professionals,^13 14^ perceptions of the pharmacist role^15–17^ and pharmacist-led care for asthma.^10 18–20^

Participants also completed an online questionnaire on demographic details (gender and age) and asthma history (self-reported asthma severity, hospitalisations and GP visits). An online questionnaire was used because participants may have felt uncomfortable disclosing personal information (eg, age) directly to a researcher during the telephone call. The self-report method was chosen because recruitment to the study may have been difficult if access to participants’ medical records was required.

All interviews were professionally transcribed, with transcripts checked for accuracy by MAM. Data were analysed using NVivo (QSR, V.11). Thematic analysis was used to identify themes at the semantic level using a deductive approach, based on their relevance to the study aim.^21^ Continuous iterative analyses were conducted to establish when thematic saturation had been reached. Recruitment was set for 30 participants or thematic saturation, whichever was attained first. The study ran from October 2017 to February 2018. All transcripts were analysed by MAM, and four transcripts (representing 25% of the total transcripts) were independently second-coded by another researcher (CBK), as recommended by MacPhail *et al*.^22^ Discrepancies were resolved through consensus discussion.

### Patient and public involvement

All study materials (recruitment flyer, participant information sheet, consent form, interview topic guide, GPP consultation example and online questionnaire) were reviewed by the AUKCAR Patient Advisory Group (PAG) prior to study commencement. Members of the PAG were adults with asthma. Their feedback, often regarding word choice and text length, was incorporated into the study materials.

## Results

Thematic saturation was reached with 17 participants (table 1). The median interview length was 42 min (ranging from 30 to 58 min). The participant sample was 59% female, with most participants (41%) aged 30 to 39 years and recruited through the Asthma UK newsletter (41%). The sample included participants with self-reported mild (53%), moderate (24%) and severe asthma (17%). Three overarching themes (with seven subthemes) were identified from the data: building trust in pharmacists, filling gaps in current asthma care and navigating a strained healthcare system.

**Table 1:** Demographic characteristics and asthma history

Characteristics (n=17)	Frequency, n (%)
Gender	
Female	10 (59)
Male	7 (41)
Age in years	
18–29	5 (29)
30–39	7 (41)
40–49	2 (12)
50–59	–
60–69	2 (12)
70+	1 (6)
Recruitment channel	
Asthma UK newsletter	7 (41)
Hospital	1 (6)
Social media	3 (17)
University College London newsletter	3 (18)
Word of mouth	3 (18)
Self-reported asthma severity	
Mild	9 (53)
Moderate	4 (24)
Severe	3 (17)
Prefer not to disclose	1 (6)
Self-reported hospitalisations for asthma (previous 12 months)	
0	11 (65)
1–4	3 (17)
5–10	3 (17)
Self-reported GP visits for asthma (previous 12 months)	
0	2 (12)
1–10	13 (76)
10–20	2 (12)

GP, general practitioner.

### Theme 1: building trust in pharmacists

Trust in healthcare professionals involves the optimistic acceptance of being in a vulnerable situation, knowing that one’s interests will be cared for.^14^ For participants, opinions of the new service were based on the level of trust they placed in pharmacists. Trust was built over time, based on perceived clinical competency and through a benchmarking process, which form the subthemes discussed below.

#### Building trust over time

Participants highlighted that trust in any healthcare professional builds through consistent contact over time. Some participants felt hesitant about the new service because it meant deviating from their usual trusted healthcare professional, suggesting a preference for usual care over new initiatives to maintain the quality of their asthma care.

‘I don’t really know, I think I’d prefer a doctor (to talk to about my asthma). It’s the way it’s always been.’

– P15, male, 30–39 years, mild asthma

#### Building trust based on perceived clinical competency

When asked about specific criteria for trust, participants discussed pharmacists’ clinical competency. This included pharmacists’ asthma-specific and broad clinical knowledge. Support for the new service was high when pharmacists were viewed as knowledgeable.

‘So I know that in the pharmacy role they’re very knowledgeable. So if (adherence support for asthma) is something they want to do then why not? I have a lot of faith in somebody who’s got a lot of knowledge in something.’

– P8, female, 30–39 years, severe asthma

Some participants believed that pharmacists would need extensive additional training to develop the knowledge needed for the new service. Their main concern was that pharmacists were too medication-focused and therefore lacked broader clinical skills.

‘…it could be that the (medication) side-effects are something else entirely. So (pharmacists) would be kind of completely thinking down the asthma route, ‘it might just be that you’re taking an inhaler that you feel side-effects’…but what if it turns out you actually have cancer?’

– P17, male, 18–29 years, mild asthma

#### Building trust through a benchmarking process

None of the participants had previous experiences with GPPs, with some participants questioning the differences between GPPs and community pharmacists. Many participants engaged in a benchmarking process: using their trust and previous experiences of other healthcare professionals to gauge how much they could trust a GPP. Common reference points included community pharmacists, respiratory consultants, nurses, GPs and paramedics.

‘…I know (pharmacist support) is there but I still don’t understand it with (brittle) asthma because I still get wary. If paramedics have never heard of it and don’t know what they’re doing, how’s a pharmacist going to hear of it?’

– P2, female, 30–39 years, severe asthma

‘I would much rather go to a pharmacist than to a nurse to discuss the medication issues that I was having… I can see an asthma nurse to discuss medication, and I was like ‘Really? What do nurses know about…not to be rude, but what do they know about medication more than my specialist who prescribed it?’

– P4, female, 30–39 years, mild asthma

### Theme 2: filling gaps in current asthma care

Participants’ opinions of GPPs were also informed by perceived gaps in their current asthma care. Participants evaluated the new service’s place in their current care based on potential role overlap between GPPs and other healthcare professionals, continuity of care and medication-specific support.

#### Potential role overlap

Participants that saw potential role overlap between GPPs and other healthcare professionals were more sceptical of the new service.

‘I think if I was having an annual asthma review I wouldn’t need to use the pharmacist’s service as well, but it might be an alternative to the annual asthma review…’

– P3, male, 70+years, mild asthma

However, other participants clearly delineated the GPP role, and these participants often recommended ways to integrate pharmacists into their care.

‘If you’re asking a GP, you’ve got maybe five, ten minutes… If you know you’ve got another ten or fifteen minutes with this pharmacist… for asking all these questions…with the GP you can concentrate on the problem and get that sorted, and then go see the pharmacist and discuss the medication.’

– P5, female, 60–69 years, moderate asthma

#### Continuity of care

Participants with self-reported severe asthma often had multiple healthcare professionals involved in their care (eg, respiratory consultants, GPs and asthma nurses). When asked about the new service, some participants felt concerned about involving an additional healthcare professional in their care. This was unrelated to their views on pharmacist competency and was usually influenced by previous experiences of inadequate continuity of care due to a lack of communication between healthcare professionals.

‘(Pharmacists) always say speak to your GP but then the GP tells you to speak to the pharmacist because they’re supposed to know more about drugs than what they are…and then you’re somewhere in the middle…’

– P2, female, 30–39 years, severe asthma

#### Medication-specific support

Other participants with severe asthma who spoke about being on multiple medications and/or having other health concerns welcomed the service. This enthusiasm came from the fact that they identified gaps in their current care that they believed could be filled by pharmacists as medication experts.

‘…just having contact with someone who actually…knows about the medication, like they know how they work and what the potential side effects are going to be and interactions…it’s that knowledge that a GP wouldn’t necessarily have time to tell you all about…’

– P6, female, 30–39 years, severe asthma

‘…I’m trying to conceive at the moment so…and I thought I don’t want to be taking anything that’s unnatural or steroid-y…I did ask the respiratory consultant (about asthma medications and In Vitro Fertilisation) but he didn’t know…’

–P8, female, 30–39 years, severe asthma

Some participants felt that GPPs should have an independent prescribing qualification to fulfil their role as medication experts. They worried that the new service might contribute to the burden on patients and/or the healthcare system and that independent pharmacist prescribers would minimise the risk of this happening.

‘For me, it would just be down to whether or not (pharmacists) are able to prescribe. I don’t imagine that they wouldn’t have the knowledge that was required…It’s just if I had to then see a doctor to be prescribed a different medication, I’d rather just go to see the doctor instead.’

– P10, female, 40–49 years, mild asthma

### Theme 3: navigating a strained healthcare system

Participants were acutely aware of the limited resources within general practice. They often expressed guilt and frustration about booking appointments for asthma. Participants never booked appointments just for medication-related questions, and their medication-related concerns were frequently left unaddressed because other topics took priority in a consultation, particularly if the participant had multiple comorbidities. The pharmacist-led service was welcomed by these participants because they felt pharmacists would have more time to focus on their medication.

‘…come Monday morning I wouldn’t want to call the GP because I know on Monday morning they’re very, very busy…I’ll just sort of crack on at home, multi-dosing salbutamol and seeing what happens.’

– P8, female, 30–39 years, severe asthma

‘(GPs) just want you in and out… ‘oh yes, I wanted to ask you something else’ but too late now, you’re away. That’s how you feel.’

–P1, female, 30–39 years, mild asthma

#### The hierarchy of healthcare professionals

Many participants constructed a hierarchy of healthcare professionals with GPs at the top, followed by nurses and finally pharmacists. This hierarchy determined the importance of each healthcare professional’s time. Severe health concerns justified booking a GP appointment, while non-urgent concerns were viewed as more suitable for pharmacists.

The hierarchy of healthcare professionals affected opinions of GPPs in both directions. Some participants were enthusiastic about the new service because they believed it would lessen the workload of GPs and nurses. For these participants, seeing a pharmacist (the healthcare professional further down the hierarchy) felt less intimidating and formal, slightly easing concerns about taking up valuable appointment time.

‘…to be honest, GPs have bigger problems to deal with…[they’re] dealing with people with, you know, life threatening illnesses, then actually seeing the standard case of asthma or an asthma check-up isn’t the best use of (their) time.’

– P16, male, 18–29 years, moderate asthma

‘It feels less formal, I think, when you’re with a pharmacist than when you’re in the doctor’s…sometimes when you go to the doctor’s, you’re kind of clock watching…’

– P10, female, 40–49 years, mild asthma

Others felt that pharmacists could not extend into a clinical role similar to GPs and nurses, with some suggesting a triage-like function to safeguard GP time.

‘I never feel as though a pharmacist is a nurse, if you see what I mean. A nurse has practical hands-on experience of trying to make people better. The pharmacist is one who deals with the theory of medication.’

–P9, male, 60–69 years, undisclosed asthma severity

‘… the pharmacist has seen you and if there’s communication between the pharmacist and the GP, so that I guess it would help the GPs prioritise who they saw…’

–P11, female, 40–49 years, moderate asthma

However, pharmacists themselves were also viewed as a limited resource. Many participants supported moving pharmacists from community pharmacies to general practice because they experienced inadequate care in busy community pharmacies. Others were concerned that pharmacist-led adherence support with a wide scope (ie, for multiple long-term conditions) would limit access for people with asthma.

‘If (pharmacists) weren’t running a community pharmacy, if they were linked in, if they worked within the GP surgery with a lot of time, then yes, I don’t see how (a lack of time) would be an issue’

– P12, female, 30–39 years, mild asthma

‘…my worry is if a pharmacist has to do (adherence support in general practice) for asthma, what other long-term conditions will they have to do it for?’

– P6, female, 30–39 years, severe asthma

## Discussion

This is the first in-depth exploration of the perspectives of adults with asthma on pharmacist-led adherence support in general practice. This focused exploration identified potential barriers to service uptake and has the potential to help further refine and tailor the GPP service as it is rolled out.

### Interpretation of findings

Trust played an important role in participants’ initial perspectives of GPP-led care—it was an essential component of the patient-pharmacist relationship and it informed participants’ views of pharmacists’ role in asthma care. These findings suggest that general awareness of the existence of pharmacist-led services is insufficient to encourage service uptake.

Participants based their trust in pharmacists on perceived clinical competency and comparisons with other trusted healthcare professionals (eg, GPs and nurses), guided by perceptions of pharmacists’ position in the hierarchy of healthcare professionals. These findings may suggest that explicit endorsement of GPP-led care by other trusted healthcare professionals might improve service uptake among adults with asthma. Reassurance and support from GPs and nurses may address some of the concerns raised by study participants, including those about fragmented care, pharmacists’ clinical competency and potential role overlap.

Support for GPP-led care did not seem to differ based on participants’ age, gender or self-reported asthma severity—there was a variety of views in each group of participants. However, participants who spoke about being on multiple medications and/or having additional health concerns seemed to be more open to pharmacist input, perhaps because they felt current asthma care was unable to meet these additional needs. Targeting this group of adults with asthma may improve service uptake in the future.

### Strengths and limitations

The qualitative method in this study captured the complex processes behind people’s initial opinions of GPPs. The combination of recruitment channels produced variation in the sample in terms of age, self-reported asthma severity and healthcare utilisation. Telephone-based interviews enabled recruitment across the UK without increasing participant burden, thus increasing study accessibility for participants with severe asthma and limited travel capacity. In addition, telephone-based interviews can produce data of higher quality compared with face-to-face interviews when sensitive topics (eg, long-term conditions) are discussed.^23^

The participant sample may however not have captured the views of all adults with asthma because it consisted primarily of people with self-reported mild asthma (53%) and people recruited through Asthma UK (41%). Thematic saturation may have been reached due to the relative homogeneity of the participant sample. The participants recruited through Asthma UK may have had a stronger interest in asthma care or pharmacist-led support and may therefore have been more supportive of the new service compared with the general population with asthma. However, if scepticism of the new service exists among people who are more engaged in their care, then the findings may be amplified in the general population with asthma who may have less interest in asthma care.

A major drawback of the study is that none of the participants had experienced a GPP consultation directly, with some participants recruited from Scotland and Northern Ireland where the GPP scheme does not exist. While the consultation description that participants were asked to read was based on real work by a clinical respiratory pharmacist working in general practice, study findings can only be used to understand patients’ initial views of a GPP-led service. These participants represent the general population with asthma who would initially need to be convinced to engage with the service. Addressing some of the concerns identified by participants may help improve the uptake of existing GPP-led care among adults with asthma. However, participants’ views may change over time and could potentially be influenced by direct personal experience with a GPP consultation.

### Comparison with existing literature

There is limited research on patient perspectives of GPPs because the care model is relatively new. However, findings from this study align with those from community pharmacy-based research, suggesting that people with asthma may not differentiate between pharmacy sectors.

Findings align with work by Gidman *et al*,^13^ who found that people were hesitant about deviating from their usual trusted care model (often a GP). However, two of the older participants in this study were open to GPP-led care, in contrast to previous research that suggests that older patients may be less likely to accept an expanded pharmacist role.^24^

In line with the present study’s findings, previous research with general members of the public and people with asthma found that other trusted healthcare professionals (eg, GPs and nurses) were used as benchmarks to inform opinions of community pharmacist-led services.^13 20^ Similarly, Naik Panvelkar *et al* 20 found that previous positive experiences with community pharmacists raised expectations for other pharmacist-led services in a population of people with asthma.

Participants’ views of the gaps in their current asthma care shaped their perspectives of GPPs. Similarly, Boyd *et al* 25 found that recipients of the New Medicine Service welcomed pharmacists’ recommendations if they addressed a concern directly raised by the patient. While asthma care guidelines recommend a multidisciplinary approach in treating difficult asthma, the present study suggests that some people with self-reported severe asthma were hesitant to include another healthcare professional due to issues with continuity of care.^26^ In line with previous research, the hierarchy of healthcare professionals influenced perspectives of pharmacists expanding further into clinical roles.^13 15^ However, this study also found that the hierarchy increased support for pharmacist-led care to reduce the burden on GPs/nurses.

### Implications for research and practice

As the GPP model is rolled out, future studies could be conducted using in-depth interviews with people with asthma after they have experienced a GPP-led consultation. These interviews could establish if interpersonal factors (ie, rapport with the pharmacist) have an impact on patient perspectives. Ethnographic observations of pharmacist-led consultations and the general practice team will help assess pharmacists’ integration and its effect on continuity of care for asthma patients. Future recruitment should aim for greater variation in participants (eg, self-reported asthma severity) through various recruitment channels, with additional efforts to look for discordant voices when thematic saturation is reached.

Findings from this study could be implemented in efforts to increase service uptake among people with asthma. Given the benchmarking process used to establish trust in pharmacists, comparisons between GPPs and other healthcare professionals could be used to inform and engage the public. For example, public campaigns highlighting the differences and similarities between GPs and GPPs may help the public differentiate the pharmacist role and understand the added value of the new service within asthma care.

Participants wanted GPPs to have broad clinical skills and a prescribing qualification. Encouragingly, the Centre for Pharmacy Postgraduate Education has already included these components in their GPP training pathways.^27^

Although the hierarchy of healthcare professionals sometimes prevented pharmacists from being perceived as clinicians, it also made GPP appointments appear less formal and intimidating to access. Participants felt more comfortable making an appointment with a pharmacist for medication-related questions. This is encouraging because the new service may encourage people with asthma to address medication-related concerns that may be barriers to medication adherence.^28^

While the perspectives of people with asthma explored in this study show that the GPP model has promise, they identified several barriers to optimal patient engagement and service implementation that will need to be addressed for the service to be effective. Meeting patient expectations will be the first crucial step in ensuring the programme’s long-term benefit and reducing the pressure on general practice in England.

## Supplementary Material

Reviewer comments

Author's manuscript

## References

[1] World Health Organization Primary health care - now more than ever. Geneva: World Health Organization, 2008.

[2] NHS England General practice forward view. England: NHS, 2016 https://www.england.nhs.uk/gp/gpfv/

[3] Mann C, Anderson C, Avery AJ, et al Clinical pharmacists in general practice: pilot scheme. University of Nottingham, UK: NHS England, 2018 https://www.nottingham.ac.uk/pharmacy/documents/generalpracticeyearfwdrev/clinical-pharmacists-in-general-practice-pilot-scheme-full-report.pdf

[4] NHS England Clinical pharmacists in general practice pilot. England, UK: NHS England, 2015 https://www.england.nhs.uk/gp/gpfv/workforce/building-the-general-practice-workforce/cp-gp/

[5] Bårnes CB, Ulrik CS Asthma and adherence to inhaled corticosteroids: current status and future perspectives. Respir Care 2015;60:455–68. 10.4187/respcare.03200 25118311

[6] Murphy AC, Proeschal A, Brightling CE, et al The relationship between clinical outcomes and medication adherence in difficult-to-control asthma: table 1. Thorax 2012;67:751–3. 10.1136/thoraxjnl-2011-201096 22436168

[7] Cumella A Falling through the gaps: why more people need basic asthma care. United Kingdom: Asthma UK, 2017 https://www.asthma.org.uk/about/media/news/press-release-around-800000-brits-with-asthma-getting-poor-care/

[8] Mes MA, Katzer CB, Chan AHY, et al Pharmacists and medication adherence in asthma: a systematic review and meta-analysis. Eur Respir J 2018;52. doi:10.1183/13993003.00485-2018. [Epub ahead of print: 23 Aug 2018]. 29976652

[9] Elliott RA, Boyd MJ, Salema N-E, et al Supporting adherence for people starting a new medication for a long-term condition through community pharmacies: a pragmatic randomised controlled trial of the new medicine service. BMJ Qual Saf 2016;25:747–58. 10.1136/bmjqs-2015-004400 26647412

[10] Latif A, Pollock K, Boardman HF The contribution of the medicines use review (Mur) consultation to counseling practice in community pharmacies. Patient Educ Couns 2011;83:336–44. 10.1016/j.pec.2011.05.007 21621943 PMC3145978

[11] Gann B Understanding and using health experiences: the policy landscape : Ziebland S, Coulter A, Calabrese JD, Understanding and using health experiences: improving patient care. Oxford, United Kingdom: Oxford University Press, 2013.

[12] O’Brien BC, Harris IB, Beckman TJ, et al Standards for reporting qualitative research: a synthesis of recommendations. Acad Med 2014;89:1245–51. 10.1097/ACM.0000000000000388 24979285

[13] Gidman W, Ward P, McGregor L Understanding public trust in services provided by community pharmacists relative to those provided by general practitioners: a qualitative study. BMJ Open 2012;2:e000939 10.1136/bmjopen-2012-000939 PMC3358628 22586286

[14] Hall MA, Dugan E, Zheng B, et al Trust in physicians and medical institutions: what is it, can it be measured, and does it matter? Milbank Q 2001;79:613–39. 10.1111/1468-0009.00223 11789119 PMC2751209

[15] Latif A, Boardman HF, Pollock K Understanding the patient perspective of the English community pharmacy medicines use review (Mur). Res Social Adm Pharm 2013;9:949–57. 10.1016/j.sapharm.2013.01.005 23506650

[16] Tinelli M, Ryan M, Bond C Patients’ preferences for an increased pharmacist role in the management of drug therapy. Int J Pharm Pract 2009;17:275–82. 10.1211/ijpp.17.05.0004 20214269

[17] Latif A, Waring J, Watmough D, et al Examination of England’s new medicine service (NmS) of complex health care interventions in community pharmacy. Res Social Adm Pharm 2016;12:966–89. 10.1016/j.sapharm.2015.12.007 26806858

[18] Bereznicki B, Peterson G, Jackson S, et al Perceived feasibility of a community pharmacy-based asthma intervention: a qualitative follow-up study. J Clin Pharm Ther 2011;36:348–55. 10.1111/j.1365-2710.2010.01187.x 21545614

[19] Bradley F, Wagner AC, Elvey R, et al Determinants of the uptake of medicines use reviews (MURs) by community pharmacies in England: a multi-method study. Health Policy 2008;88:258–68. 10.1016/j.healthpol.2008.03.013 18468713

[20] Naik Panvelkar P, Armour C, Saini B Community pharmacy-based asthma services--what do patients prefer? J Asthma 2010;47:1085–93. 10.3109/02770903.2010.514638 21039206

[21] Braun V, Clarke V Using thematic analysis in psychology. Qual Res Psychol 2006;3:77–101. 10.1191/1478088706qp063oa

[22] MacPhail C, Khoza N, Abler L, et al Process guidelines for establishing Intercoder reliability in qualitative studies. Qual Res 2016;16:198–212. 10.1177/1468794115577012

[23] Sturges JE, Hanrahan KJ, Telephone C Comparing telephone and face-to-face qualitative interviewing: a research note. Qual Res 2004;4:107–18. 10.1177/1468794104041110

[24] Perepelkin J Public opinion of pharmacists and pharmacist prescribing. Can Pharm J 2011;144:86–93. 10.3821/1913-701X-144.2.86

[25] Boyd MJ, Elliott R, Barber N, et al The impact of the new medicines service (NmS) in England on patients adherence to their medicines. Int J Pharm Pract 2014;22.

[26] British Thoracic Society/Scottish Intercollegiate Guidelines Network British guideline on the management of asthma: a national clinical guideline. Edinburgh: British Thoracic Society/Scottish Intercollegiate Guidelines Network, 2016 https://www.brit-thoracic.org.uk/quality-improvement/guidelines/asthma

[27] Centre for Pharmacy Postgraduate Education Clinical pharmacists in general practice education. Manchester, United Kingdom: University of Manchester, 2018 https://www.cppe.ac.uk/career/cpgpe/clinical-pharmacists-in-general-practice-education

[28] Horne R, Weinman J Self-Regulation and self-management in asthma: exploring the role of illness perceptions and treatment beliefs in explaining non-adherence to preventer medication. Psychol Health 2002;17:17–32. 10.1080/08870440290001502

