# Supplementary material for: Pharmacist-led adherence support in general practice: a qualitative interview study of adults with asthma
Source: BMJ Open. 2019 Nov 6;9(11):e032084. doi: 10.1136/bmjopen-2019-032084 (PMC6858226; doi:10.1136/bmjopen-2019-032084)
Supplement: Supplementary data [file bmjopen-2019-032084supp001.pdf]

APPENDIX A: DESCRIPTION OF A PHARMACIST-LED ADHERENCE CONSULTATION DELIVERED IN GENERAL PRACTICE

We will describe this service as if you were receiving it.

The service

The service is delivered by a clinical pharmacist based at your local GP practice. The aim is to **help people with asthma get on better with their inhalers**. This means answering all of your questions and discussing all of your concerns about your inhalers in detail.

Pre-consultation

You are booked in for a consultation with the clinical pharmacist by the GP receptionist (just like you would with a GP appointment).

The main consultation

You have a one-to-one consultation with the pharmacist in a consultation room. They will begin with a **standard asthma review**. You will talk about your asthma control, recent symptoms, smoking history, and your asthma action plan. They will check your inhaler technique and lung function.

You will then have an **in-depth discussion about your inhalers**. You can ask any questions or mention any concerns you have about your inhalers. This could be anything from concerns about side effects or questions about how the medication works. The pharmacist will give you useful information and feedback that is **specifically suited to you**. They may use a short video or print-outs to guide their discussion with you.

At the end of the consultation, you will set an asthma-related goal with the pharmacist. This could be something like “less night time asthma symptoms”. The pharmacist will advise you on how to achieve your personal goal using your inhalers.

The follow-up consultation

1 month later, you will see the pharmacist again for a short consultation (no more than 10 minutes). The pharmacist will briefly ask how your asthma has been and check your inhaler technique/lung function. You will then discuss how you are getting on with your medicines and your personal asthma goal. Based on this discussion, the pharmacist will give further recommendations regarding your medication.

Mes MA, *et al. BMJ Open* 2019; 9:e032084. doi: 10.1136/bmjopen-2019-032084

APPENDIX B: INTERVIEW TOPIC GUIDE

Background and Previous Experience

1. To start the interview, could you tell me when you were diagnosed with asthma?

2. What type of medications are you currently prescribed for your asthma?

   a. How has your experience been with these medications? (Prompting to see if there are concerns, side effects etc.)

3. A lot of people with asthma have questions or concerns about their inhalers or tablets. What do you do when you have any questions or concerns specifically about your asthma medication?

   a. Prompts: do you do research online? Do you talk to friends and family? Do you go talk to a healthcare professional?
   b. Could you tell me a bit about why you prefer (insert route, e.g. researching online, talking to the GP, talking to family etc.) when it comes to your medication?

4. Have you ever talked to a pharmacist about your asthma medication?

   a. If **YES**: Could you walk me through what happened during that interaction?
      i. Where did it take place? (community pharmacist, hospital pharmacist etc.)
      ii. Prompts: what type of information did they give you? Were they able to answer your questions? Did you feel comfortable talking to them about your asthma medication? Did you feel like it was helpful for you?
      iii. Would you consider the pharmacist as a regular part of your asthma care team? Why or why not?

   b. If **NO**: Could you imagine a pharmacist as part of your regular asthma care team?

5. I'd now like to talk specifically about your preventer inhaler (brown inhaler, steroid inhaler). They're normally prescribed for daily or twice daily use. A lot of asthma patients we've talked to often don't take the inhaler as regularly as prescribed, is this something you've experienced as well?
   a. Could you tell me a little bit about why?

Pharmacist in general practice

Great, that's the first part of our interview. Now we're going to continue on to the part where I'd like to get your thoughts on pharmacist-led consultations in general practice. I've e-mailed you a description of the service already, but would you like to review it to refresh your memory?

Mes MA, *et al. BMJ Open* 2019; 9:e032084. doi: 10.1136/bmjopen-2019-032084

Do you have any questions or anything you would like me to clarify before we move on to the questions?

6. What are your initial thoughts on this pharmacist medication support service?
    a. Explore which aspects of the service are most salient to the participant, use prompts below when needed.

- In your opinion, would this type of service be a useful addition to the care you get from your GP/nurse/asthma specialist?
    - Is this a type of service you could see yourself using? Why or why not?
    - Do you think this service is any different from the asthma care you already receive?
- Do you think this type of service would help you take your preventer inhaler more regularly?
- How do you feel about a pharmacist taking on this type of clinical role?
    - Do you think pharmacists have enough training for this type of work?
        - What type of knowledge would you expect the pharmacist to have?
    - How do pharmacists compare to your other healthcare professionals (e.g. GP or nurse)?
    - Do you think this service is any different from seeing a community pharmacist? (Are they familiar with the NMS and MUR?)
    - Would you feel comfortable talking to a pharmacist about your asthma and your medication?
    - Do you feel like you can trust a pharmacist in this type of role?
- How do you feel about having a pharmacist based in a GP surgery?
    - How would you feel about this pharmacist having access to your medical records?
- What do you think about seeing a pharmacist on an appointment basis?
- When you think about (concerns/side effects discussed for question 2a), do you think those types of questions or concerns could be addressed by a pharmacist?
    - What would be the ideal way to support you with those questions or concerns?
- Do you think this type of service is convenient for people with asthma?

Mes MA, *et al. BMJ Open* 2019; 9:e032084. doi: 10.1136/bmjopen-2019-032084
