# Supplementary material for: Pharmacist-led adherence support in general practice: a qualitative interview study of adults with asthma
Source: BMJ Open. 2019 Nov 6;9(11):e032084. doi: 10.1136/bmjopen-2019-032084 (PMC6858226; doi:10.1136/bmjopen-2019-032084)
Supplement: Author's manuscript [file bmjopen-2019-032084.draft_revisions.pdf]

BMJ Open

BMJ Open is committed to open peer review. As part of this commitment we make the peer review history of every article we publish publicly available.

When an article is published we post the peer reviewers' comments and the authors' responses online. We also post the versions of the paper that were used during peer review. These are the versions that the peer review comments apply to.

The versions of the paper that follow are the versions that were submitted during the peer review process. They are not the versions of record or the final published versions. They should not be cited or distributed as the published version of this manuscript.

BMJ Open is an open access journal and the full, final, typeset and author-corrected version of record of the manuscript is available on our site with no access controls, subscription charges or pay-per-view fees (http://bmjopen.bmj.com).

If you have any questions on BMJ Open's open peer review process please email info.bmjopen@bmj.com

Pharmacist-led adherence support in general practice: a qualitative interview study of adults with asthma

Journal:	*BMJ Open*
Manuscript ID	bmjopen-2019-032084
Article Type:	Research
Date Submitted by the Author:	01-Jun-2019
Complete List of Authors:	Mes, Marissa; UCL School of Pharmacy, Centre for Behavioural Medicine, Practice & Policy<br>Katzer, Caroline; UCL School of Pharmacy, Centre for Behavioural Medicine, Practice & Policy<br>Wileman, Vari; UCL School of Pharmacy, Centre for Behavioural Medicine, Practice & Policy<br>Chan, Amy; UCL School of Pharmacy, Centre for Behavioural Medicine, Practice & Policy<br>Horne, Robert; UCL School of Pharmacy, Centre for Behavioural Medicine, Practice & Policy<br>Taylor, Stephanie; Queen Mary University of London, Centre for Primary Care and Public Health
Keywords:	Medication Adherence, Asthma < THORACIC MEDICINE, Pharmaceutical Services, General Practice, Medication Therapy Management

SCHOLARONE™
Manuscripts

Pharmacist-led adherence support in general practice: a qualitative interview study of adults with asthma

Mes, Marissa Ayano[1]; Katzer, Caroline Brigitte[1]; Wileman, Vari[1]; Chan, Amy Hai Yan[1]; Horne, Rob[1]; & Taylor, Stephanie Jane Caroline[2].

[1]Centre for Behavioural Medicine, UCL School of Pharmacy, Mezzanine Floor, Tavistock Square, London WC1H 9JP, London, United Kingdom.

[2]Centre for Primary Care and Public Health, Blizard Institute, Queen Mary University London, Yvonne Carter Building, 58 Turner Street, London E1 2AB, United Kingdom.

Corresponding author: Marissa Ayano Mes (marissa.mes.15@ucl.ac.uk, +44 7413525215), Centre for Behavioural Medicine, UCL School of Pharmacy, Mezzanine Floor, Tavistock Square, London WC1H 9JP, United Kingdom.

Word count: 3,902 words

Key words: Medication Adherence, Asthma, Pharmaceutical Services, General Practice, Medication Therapy Management

For peer review only

ABSTRACT

Objectives: The National Health Service (NHS) in England recently introduced general practice pharmacists (GPPs) to provide medication-focused support to both patients and the general practice team. This healthcare model may benefit people with asthma, who currently receive sub-optimal care and demonstrate low medication adherence. This study aimed to explore the perspectives of adults with asthma on pharmacist-led adherence support delivered in general practice, with a focus on how these perspectives are formed.

Design and setting: The study was conducted in the United Kingdom (UK) utilising a qualitative interview methodology. Participants completed a telephone-based semi-structured interview, followed by an online questionnaire for demographic details and asthma history. Qualitative data were analysed using thematic analysis.

Participants: Participants (n = 17) were adults with asthma in the UK with a prescription for an inhaled corticosteroid. Participants did not have previous experience with GPPs and were asked to provide their views on a proposed GPP-led service.

Results: Participant perspectives of GPPs were determined by trust in pharmacists, perceived gaps in asthma care, and the perceived strain on the NHS. Trust was based on pharmacists' perceived clinical competency, established over time, and gauged through a 'benchmarking' process. GPP's fit in current asthma care was assessed based on potential role overlap with other healthcare professionals, continuity of care, and medication-related support needs. Participants navigated the NHS based on a perceived hierarchy of healthcare professionals (GPs on top, nurses, then pharmacists), and this influenced their perspectives of GPPs.

Conclusions: While the GPP scheme shows promise based on the perspectives of people with asthma, the identified barriers to optimal patient engagement and service implementation will need to be addressed for the service to be effective.

For peer review only - http://bmjopen.bmj.com/site/about/guidelines.xhtml

ARTICLE SUMMARY

strengths and limitations of this study

- The use of qualitative methodology captured the complex processes behind people's perceptions of general practice pharmacists (GPPs), including how lived experiences shaped perceptions of new pharmacist-led services.
- Telephone-based interviews enabled recruitment across the United Kingdom (UK) and increased study accessibility for people with severe asthma and travel limitations.
- The study may not have captured the full range of views among adults with asthma in the UK because participants were primarily white and female.
- Participants had no experience with GPP-led consultations and therefore represented the general population with asthma that would initially need to be convinced to engage with the service.

INTRODUCTION

The pressure on primary care to deliver core services is increasing rapidly worldwide due to a growing and ageing population, the prevalence of long-term conditions, and significant resource constraints. Primary care systems are being reshaped and new models of care are emerging to cope with growing demand.[1]

One such model is the general practice pharmacist (GPP) model, which was introduced in England as part of the NHS *General Practice Forward View* initiative.[2-4] These pharmacists support both patients and the general practice team with medication-related issues, with the aim of expanding the general practice workforce, reducing practice burden, and increasing patient access to appointments.[4] Initial qualitative feedback from general practitioners (GPs) and pharmacists in a pilot study in England suggests that GPPs can have positive impacts on

For peer review only - http://bmjopen.bmj.com/site/about/guidelines.xhtml

medication safety, medication adherence, healthcare access, and patient satisfaction across a variety of long-term conditions.[3]

A long-term condition such as asthma may benefit from GPP support. Research shows that adherence to inhaled corticosteroids (ICS), an essential medication for asthma, is low.[5 6] Furthermore, only 35% of people with asthma in the United Kingdom (UK) receive basic care (i.e. annual reviews, inhaler technique checks, and a written asthma action plan).[7] Previous research suggests that pharmacist-led interventions can increase medication adherence and support asthma self-management among people with asthma.[8] However, most of the previous research in the UK has been limited to community rather than general practice pharmacists, and was focused on their impact across a range of long-term conditions rather than asthma specifically.[9 10]

Understanding the specific perspectives of people with asthma regarding GPPs is an important first step in establishing the potential benefits of this new service for this specific patient group and identifying any potential issues in its future uptake and effectiveness.[11] The aim of this study was to explore the perspectives of adults with asthma on pharmacist-led asthma adherence support delivered in general practice, with a focus on how these perspectives are formed.

METHODS

This was a telephone-based semi-structured interview study, using an interpretivist approach to understand how adults with asthma construct their perceptions of GPP-led asthma adherence support. Demographic details and asthma history were collected using a brief online questionnaire.

For peer review only - http://bmjopen.bmj.com/site/about/guidelines.xhtml

participants

Participants were adults (≥ 18 years old) living in the UK and proficient in English, with a self-reported asthma diagnosis, a prescription for ICS, and access to a telephone and e-mail account. People with respiratory comorbidities (e.g. Chronic Obstructive Pulmonary Disease) and/or those in hospital or nursing homes were excluded, as the adherence behaviour and support needs of these individuals were hypothesised to be different.

recruitment

Several recruitment channels were used to ensure that participants varied in age, gender, and self-reported asthma severity. A flyer with study information and researchers' contact details was circulated by researchers, the Asthma UK Centre for Applied Research (AUKCAR), and the National Institute for Health Research Collaboration for Leadership in Applied Health Research and Care (NIHR CLAHRC) North Thames via social media (Facebook and/or Twitter). The study was advertised in two electronic newsletters: the Asthma UK volunteer bulletin and the University College London student newsletter. Printed flyers were handed directly to potential participants by a respiratory consultant at a London hospital, and pharmacists at two hospitals in Wales.

People who contacted the researchers were e-mailed an information sheet, consent form, and eligibility criteria to review. If they were eligible and willing to participate, they were booked in for a one-hour telephone interview. In preparation for the call, participants were asked to read a description of a GPP-led adherence support consultation, sent to them via e-mail (see Appendix A). This description was developed based on the work of a clinical respiratory pharmacist working in general practice in London. Participants gave verbal consent over the telephone before the interview began. The consent procedure and interview were audio-

For peer review only

recorded with their permission. All participants were e-mailed a £20 online shopping voucher after the interview to thank them for their time.

data collection and analysis

One researcher (MM) conducted the interviews. Participants were informed that the researcher had a background in Health Psychology and an interest in adherence and pharmacist-led care for asthma.

The topic guide for the telephone-based interview had two sections (see Appendix B). The first section focused on participants' previous experiences of asthma, asthma care, and pharmacists. The second section focused on how these lived experiences informed participants' perceptions of GPP-led adherence support. Questions in the second section were based on previous research on interpersonal and institutional trust in healthcare professionals,[12 13] perceptions of the pharmacist role,[14-16] and pharmacist-led care for asthma.[10 17-19]

Participants also completed an online questionnaire on demographic details and asthma history (self-reported asthma severity, hospitalisations, and GP visits). An online questionnaire was used because disclosing personal information (e.g. ethnicity or age) directly to a researcher during a telephone call can be uncomfortable for some participants. The self-report method was chosen because requesting access to people's medical records can feel invasive for some participants and can therefore hinder recruitment.

All interviews were transcribed and analysed using NVivo (QSR, Version 11). Thematic analysis was used to identify themes at the semantic level using a deductive approach, based on their relevance to the study aim.[20] The flexibility of the thematic analysis method was deemed a suitable fit for the study's exploratory nature. Continuous iterative analyses were conducted to establish when thematic saturation had been reached. Recruitment was set for

For peer review only - http://bmjopen.bmj.com/site/about/guidelines.xhtml

30 participants or thematic saturation, whichever was attained first. All transcripts were analysed by MM, and 25% of the transcripts were independently second-coded by another researcher (CK). Discrepancies were resolved through consensus discussion.

patient and public involvement

All study materials (the recruitment flyer, participant information sheet, consent form, interview topic guide, GPP consultation example, and online questionnaire) were reviewed by the AUKCAR Patient Advisory Group (PAG) prior to study commencement. Members of the PAG were adults with asthma. Their feedback, often regarding word choice and text length, was incorporated into the study materials.

RESULTS

Thematic saturation was reached with 17 participants (Table 1). The mean interview length was 39 minutes (ranging from 30 to 58 minutes). The participant sample was mostly female (59%), aged 30 to 39 years (41%), and White-British (70%). Most participants were recruited through the Asthma UK newsletter (41%). The sample included participants with self-reported mild (53%), moderate (24%), and severe asthma (17%). Three overarching themes (with seven sub-themes) were identified from the data: building trust in pharmacists, filling gaps in current asthma care, and navigating a strained healthcare system.

Table 1. Demographic characteristics and asthma history

**Characteristics** (n = 17)	**Frequency** n (%)
**Gender**	
Female	10 (59%)
Male	7 (41%)
**Age in years**	
18 – 29	5 (29%)
30 – 39	7 (41%)
40 – 49	2 (12%)
50 - 59	-
60 – 69	2 (12%)
70 +	1 (6%)

For peer review only - http://bmjopen.bmj.com/site/about/guidelines.xhtml

**Ethnicity**	
Black or Black British	1 (6%)
Mixed – White and Black African	1 (6%)
White – British	12 (70%)
White – Irish	1 (6%)
White – Any other background	2 (12%)
**Location of residence**	
England – North	1 (6%)
England – Midlands	2 (12%)
England – South	7 (41%)
Northern Ireland	1 (6%)
Scotland	6 (35%)
**Recruitment channel**	
Asthma UK newsletter	7 (41%)
Hospital	1 (6%)
Social media	3 (17%)
University College London newsletter	3 (18%)
Word of Mouth	3 (18%)
**Self-reported asthma severity**	
Mild	9 (53%)
Moderate	4 (24%)
Severe	3 (17%)
Prefer not to disclose	1 (6%)
**Self-reported hospitalisations for asthma (previous 12 months)**	
0	11 (65%)
1 – 4	3 (17%)
5 – 10	3 (17%)
**Self-reported GP visits for asthma (previous 12 months)**	
0	2 (12%)
1 – 10	13 (76%)
10 – 20	2 (12%)

theme 1: building trust in pharmacists

Trust in healthcare professionals involves the optimistic acceptance of being in a vulnerable situation, knowing that one's interests will be cared for.[13] For participants, opinions of the new service were based on the level of trust they placed in pharmacists. Trust was built over time, based on perceived clinical competency, and through a benchmarking process, which form the subthemes discussed below.

For peer review only - http://bmjopen.bmj.com/site/about/guidelines.xhtml

Building trust over time

Participants highlighted that trust in any healthcare professional builds through consistent contact over time. Some participants felt hesitant about the new service because it meant deviating from their usual trusted healthcare professional, suggesting a preference for usual care over new initiatives to maintain the quality of their asthma care.

"I don't really know, I think I'd prefer a doctor [to talk to about my asthma]. It's the way it's always been."
– P15, male, 30 – 39 years, mild asthma

Building trust based on perceived clinical competency

When asked about specific criteria for trust, participants discussed pharmacists' clinical competency. One element of competency was pharmacists' asthma-specific and broad clinical knowledge. Support for the new service was high when pharmacists were viewed as knowledgeable.

"So I know that in the pharmacy role they're very knowledgeable. So if [adherence support for asthma] is something they want to do then why not? I have a lot of faith in somebody who's got a lot of knowledge in something."
– P8, female, 30 – 39 years, severe asthma

Some participants believed that pharmacists would need extensive additional training to develop the knowledge needed for the new service. Their main concern was that pharmacists were too medication-focused and therefore lacked broader clinical skills.

"…it could be that the [medication] side-effects are something else entirely. So [pharmacists] would be kind of completely thinking down the asthma route, 'it might just be that you're taking an inhaler that you feel side-effects'…but what if it turns out you actually have cancer?"
– P17, male, 18 – 29 years, mild asthma

For peer review only - http://bmjopen.bmj.com/site/about/guidelines.xhtml

Building trust through a benchmarking process

None of the participants had previous experiences with GPPs, with some participants questioning the differences between GPPs and community pharmacists. Many participants engaged in a *benchmarking* process: using their trust and previous experiences of other healthcare professionals to gauge how much they could trust a GPP. Common reference points included community pharmacists, respiratory consultants, nurses, GPs, and paramedics.

"…I know [pharmacist support] is there but I still don't understand it with [brittle] asthma because I still get wary. If paramedics have never heard of it and don't know what they're doing, how's a pharmacist going to hear of it?"
– P2, female, 30 – 39 years, severe asthma

"I would much rather go to a pharmacist than to a nurse to discuss the medication issues that I was having… I can see an asthma nurse to discuss medication, and I was like 'Really?…not to be rude, but what do [nurses] know about medication more than my specialist who prescribed it?"
– P4, female, 30 – 39 years, mild asthma

theme 2: filling gaps in current asthma care

Participants' perceptions of GPPs were also informed by perceived gaps in their current asthma care. Participants evaluated the new service's place in their current care based on potential role overlap between GPPs and other healthcare professionals, continuity of care, and medication-specific support.

Potential role overlap

Participants that saw potential role overlap between GPPs and other healthcare professionals were more sceptical of the new service. However, other participants clearly delineated the GPP role, and these participants often recommended ways to integrate pharmacists into their care.

For peer review only - http://bmjopen.bmj.com/site/about/guidelines.xhtml

"I think if I was having an annual asthma review, I wouldn't need to use the pharmacist's service as well, but it might be an alternative to the annual asthma review…"
– P3, male, 70+ years, mild asthma

"If you're asking a GP, you've got maybe five, ten minutes… If you know you've got another ten or fifteen minutes with this pharmacist… for asking all these questions…with the GP you can concentrate on the problem and get that sorted, and then go see the pharmacist and discuss the medication."
– P5, female, 60 – 69 years, moderate asthma

Continuity of care

Participants with self-reported severe asthma often had multiple healthcare professionals involved in their care (e.g. respiratory consultants, GPs, and asthma nurses). When asked about the new service, some participants felt concerned about involving an additional healthcare professional in their care. This was unrelated to their views on pharmacist competency, and was usually influenced by previous experiences of inadequate continuity of care due to a lack of communication between healthcare professionals.

"[Pharmacists] always say speak to your GP but then the GP tells you to speak to the pharmacist because they're supposed to know more about drugs than what they are…and then you're somewhere in the middle…"
– P2, female, 30 – 39 years, severe asthma

Medication-specific support

Other participants with severe asthma on multiple medications and/or with other health concerns welcomed the service. This enthusiasm came from the fact that they identified gaps in their current care which they believed could be filled by pharmacists as medication experts.

"…just having contact with someone who actually…knows about the medication, like they know how they work and what the potential side effects are going to be and interactions…it's that knowledge that a GP wouldn't necessarily have time to tell you all about…"
– P6, female, 30 – 39 years, severe asthma

For peer review only - http://bmjopen.bmj.com/site/about/guidelines.xhtml

"…I'm trying to conceive at the moment so…and I thought I don't want to be taking anything that's unnatural or steroid-y…I did ask the respiratory consultant [about asthma medications and In Vitro Fertilisation] but he didn't know…"

–P8, female, 30 – 39 years, severe asthma

Some participants felt that GPPs should have an independent prescribing qualification to fulfil their role as medication experts. They worried that the new service might contribute to the burden on patients and/or the healthcare system, and that independent pharmacist prescribers would minimise the risk of this happening.

"For me, it would just be down to whether or not [pharmacists] are able to prescribe. I don't imagine that they wouldn't have the knowledge that was required…It's just if I had to then see a doctor to be prescribed a different medication, I'd rather just go to see the doctor instead."

– P10, female, 40 – 49 years, mild asthma

theme 3: navigating a strained healthcare system

Participants were acutely aware of the limited resources within general practice. They often expressed guilt and frustration about booking appointments for asthma. Participants never booked appointments just for medication-related questions, and their concerns were frequently left unaddressed because other topics took priority in a consultation, particularly if the participant had multiple comorbidities. The pharmacist-led service was welcomed by these participants because they felt pharmacists would have more time to focus on their medication.

"…come Monday morning I wouldn't want to call the GP because I know on Monday morning they're very, very busy…I'll just sort of crack on at home, multi-dosing salbutamol and seeing what happens."

– P8, female, 30 – 39 years, severe asthma

"[GPs] just want you in and out… 'oh yes, I wanted to ask you something else' but too late now, you're away. That's how you feel."

–P1, female, 30 – 39 years, mild asthma

For peer review only - http://bmjopen.bmj.com/site/about/guidelines.xhtml

The hierarchy of healthcare professionals

Many participants constructed a hierarchy of healthcare professionals with GPs at the top, followed by nurses and finally pharmacists. This hierarchy determined the importance of each healthcare professionals' time. Severe health concerns justified booking a GP appointment, while non-urgent concerns were viewed as more suitable for pharmacists. The hierarchy of healthcare professionals affected perceptions of GPPs in both directions. Some participants were enthusiastic about the new service because they believed it would lessen the workload of GPs and nurses. For these participants, seeing a pharmacist (the healthcare professional further down the hierarchy) felt less intimidating and formal, slightly easing concerns about taking up valuable appointment time.

"…to be honest, GPs have bigger problems to deal with…[they're] dealing with people with, you know, life threatening illnesses, then actually seeing the standard case of asthma or an asthma check-up isn't the best use of [their] time."
– P16, male, 18 – 29 years, moderate asthma

"It feels less formal, I think, when you're with a pharmacist than when you're in the doctor's…sometimes when you go to the doctor's, you're kind of clock watching…"
– P10, female, 40 – 49 years, mild asthma

Others felt that pharmacists could not extend into a clinical role similar to GPs and nurses, with some suggesting a triage-like function to safeguard GP time.

"I never feel as though a pharmacist is a nurse, if you see what I mean. A nurse has practical hands-on experience of trying to make people better. The pharmacist is one who deals with the theory of medication."
–P9, male, 60 – 69 years, undisclosed asthma severity

"… the pharmacist has seen you and if there's communication between the pharmacist and the GP, so that I guess it would help the GPs prioritise who they saw…"
–P11, female, 40 – 49 years, moderate asthma

However, pharmacists themselves were also viewed as a limited resource. Many participants supported moving pharmacists from community pharmacies to general practice because they experienced inadequate care in busy community pharmacies. Others were concerned that

For peer review only - http://bmjopen.bmj.com/site/about/guidelines.xhtml

pharmacist-led adherence support with a wide scope (i.e. for multiple long-term conditions) would limit access for people with asthma.

"If [pharmacists] weren't running a community pharmacy, if they were linked in, if they worked within the GP surgery with a lot of time, then yes, I don't see how [a lack of time] would be an issue."

– P12, female, 30 – 39 years, mild asthma

"…my worry is if a pharmacist has to do [adherence support in general practice] for asthma, what other long-term conditions will they have to do it for?"

– P6, female, 30 – 39 years, severe asthma

DISCUSSION

This is the first in-depth exploration of the perspectives of adults with asthma on pharmacist-led adherence support in general practice. This focused exploration identified potential barriers to service uptake and has the potential to help further refine and tailor the GPP service as it is rolled out.

main findings

Perceptions of GPPs were informed by people's trust in pharmacists, perceived gaps in current asthma care, and the perceived strain on the NHS. Trust was based on GPPs' perceived clinical competency, which was established over time, and gauged through a benchmarking process using other healthcare professionals as reference points. Community pharmacists were commonly referenced in the benchmarking process, suggesting that participants did not delineate between pharmacy sectors. Participants contemplated how GPPs would fit into their current asthma care based on potential role overlap with other healthcare professionals, issues with continuity of care, and specific medication-related support needs. The perceived strain on the NHS led participants to use a hierarchy of healthcare professionals to guide their interactions with the healthcare system. For some participants, the hierarchy categorised pharmacist consultations as informal and more

For peer review only - http://bmjopen.bmj.com/site/about/guidelines.xhtml

accessible sources of support. For others, their views acted as barriers that prevented pharmacists from extending into consultation-based roles.

strengths and limitations

The qualitative method in this study captured the complex processes behind people's perceptions of GPPs. The combination of recruitment channels produced variation in the sample in terms of age, self-reported asthma severity, healthcare utilisation, and participants' place of residence. Telephone-based interviews enabled recruitment across the UK without increasing participant burden, thus increasing study accessibility for participants with severe asthma and limited travel capacity. In addition, telephone-based interviews can produce data of higher quality compared to face-to-face interviews when sensitive topics (e.g. long-term conditions) are discussed.[21]

The participant sample may however not have captured the views of all adults with asthma because it consisted primarily of White females. Therefore, thematic saturation may have been reached due to a lack of variation in the perspectives in the sample. The participants recruited through Asthma UK (41%) may have had a strong interest in asthma care or pharmacist-led support. However, if scepticism of the new service exists among people who are more engaged in their care, then this suggests that the findings may be amplified in the general population with asthma who may have less interest in asthma care.

A major drawback of the study is that none of the participants had experienced a GPP consultation directly, with some participants recruited from Scotland and Northern Ireland where the GPP scheme does not exist. However, these participants represent the general population with asthma who would initially need to be convinced to engage with the service. Furthermore, the consultation description that participants were asked to read was based on real work by a clinical respiratory pharmacist working in general practice. These study

For peer review only - http://bmjopen.bmj.com/site/about/guidelines.xhtml

findings therefore reflect patients' initial views of a GPP service, though these views may change over time, and could potentially be influenced by direct personal experience with a GPP.

comparison with existing literature

There is limited research on patient perspectives of GPPs because the care model is relatively new. However, findings from this study align with those from community pharmacy-based research, suggesting that people with asthma may not differentiate between pharmacy sectors.

Findings align with work by Gidman, et al. [12], who found that people were hesitant about deviating from their usual trusted care model (often a GP). Both general members of the public and people with asthma have been found to use other trusted healthcare professionals (e.g. GPs and nurses) as benchmarks for trust when asked about a community pharmacist-led service.[12 19] Similarly, Naik Panvelkar, et al. [19] found that previous positive experiences with community pharmacists raised expectations for other pharmacist-led services in a population of people with asthma.

Participants' views of the gaps in their current asthma care shaped their perspectives of GPPs. Similarly, Boyd, et al. [22] found that recipients of the New Medicine Service (NMS) welcomed pharmacists' recommendations if they addressed a concern directly raised by the patient. While asthma care guidelines recommend a multidisciplinary approach in treating difficult asthma, the present study suggests that some people with self-reported severe asthma were hesitant to include another healthcare professional due to issues with continuity of care.[23] In line with previous research, the hierarchy of healthcare professionals influenced perspectives of pharmacists expanding further into clinical roles.[12 14] However, this study also found that the hierarchy increased support for pharmacist-led care to reduce the burden on GPs/nurses.

For peer review only - http://bmjopen.bmj.com/site/about/guidelines.xhtml

implications for research and practice

As the GPP model is rolled out, future studies could be conducted using in-depth interviews with people with asthma after they have experienced a GPP-led consultation. These interviews could establish if interpersonal factors (i.e. rapport with the pharmacist) have an impact on patient perspectives. Ethnographic observations of pharmacist-led consultations and the general practice team will help assess pharmacists' integration and its effect on continuity of care for asthma patients. Future recruitment should aim for greater variation in participants (e.g. age and ethnicity) through various recruitment channels, with additional efforts to look for discordant voices when thematic saturation is reached.

Findings from this study could be implemented in efforts to increase service uptake among people with asthma. Given the *benchmarking* process used to establish trust in pharmacists, comparisons between GPPs and other healthcare professionals could be used to inform and engage the public. For example, public campaigns highlighting the differences and similarities between GPs and GPPs may help the public differentiate the pharmacist role and understand the added value of the new service within asthma care.

Participants wanted GPPs to have broad clinical skills and a prescribing qualification. The Centre for Pharmacy Postgraduate Education has already included these components in their GPP training pathways, and these should be made a programme priority.[24]

Although the hierarchy of healthcare professionals sometimes prevented pharmacists from being perceived as clinicians, it also made GPP appointments appear less formal and intimidating to access. Participants felt more comfortable making an appointment with a pharmacist for medication-related questions. This is encouraging because the new service may encourage people with asthma to address medication-related concerns that may be barriers to medication adherence.[25]

For peer review only - http://bmjopen.bmj.com/site/about/guidelines.xhtml

While the perspectives of people with asthma explored in this study show that the GPP model has promise, they identified several barriers to optimal patient engagement and service implementation that will need to be addressed for the service to be effective. Meeting patient expectations will be the first crucial step in ensuring the programme's long-term benefit and reducing the pressure on general practice in England.

For peer review only

For peer review only - http://bmjopen.bmj.com/site/about/guidelines.xhtml

ACKNOWLEDGMENTS

We would like to thank the Asthma UK Centre for Applied Research (AUKCAR) Patient Advisory Group for their feedback and support for this study.

AUTHOR CONTRIBUTIONS

The study was designed by MM, RH, and SJCT. All interviews were conducted by MM. Data was analysed by MM and CK. The manuscript was written by MM, AHYC, and VW, with input from RH and SJCT.

FUNDING

The research was funded by the National Institute for Health Research (NIHR) Collaboration for Leadership in Applied Health Research and Care North Thames at Barts Health NHS Trust. The views expressed are those of the authors and not necessarily those of the NHS, the NIHR, or the Department of Health and Social Care.

COMPETING INTERESTS

None declared.

PATIENT CONSENT FOR PUBLICATION

Not required.

ETHICS APPROVAL

The research was approved by the NHS London-Harrow Research Ethics Committee (12th October 2017, Ref: 17/LO/1565) and Cwm Taf University Health Board (17th November 2017, Ref: CT/831/205928/17).

For peer review only

DATA SHARING STATEMENT

No additional data are available.

For peer review only

For peer review only - http://bmjopen.bmj.com/site/about/guidelines.xhtml

REFERENCES

1. World Health Organization. Primary Health Care - Now More Than Ever. The World Health Report. Geneva: World Health Organization, 2008.
2. NHS England. General Practice Forward View: NHS England, 2016:1-58.
3. Boyd MJ, Mann C, Waring J, et al. Clinical pharmacists in general practice: pilot scheme. Independent Evaluation Report. University of Nottingham, UK: NHS England, 2018.
4. NHS England. Clinical pharmacists in general practice pilot. England, UK: NHS England, 2015.
5. Barnes CB, Ulrik CS. Asthma and adherence to inhaled corticosteroids: current status and future perspectives. *Respir Care* 2015;60(3):455-68. doi: 10.4187/respcare.03200 [published Online First: 2014/08/15]
6. Murphy AC, Proeschal A, Brightling CE, et al. The relationship between clinical outcomes and medication adherence in difficult-to-control asthma. *Thorax* 2012;67(8):751-3. doi: 10.1136/thoraxjnl-2011-201096 [published Online First: 2012/03/23]
7. Cumella A. Falling through the gaps: Why more people need basic asthma care. Annual Asthma Survey. United Kingdom: Asthma UK, 2017.
8. Mes MA, Katzer CB, Chan AHY, et al. Pharmacists and medication adherence in asthma: a systematic review and meta-analysis. *Eur Respir J* 2018;52(2) doi: 10.1183/13993003.00485-2018 [published Online First: 2018/07/07]
9. Elliott R, Boyd MJ, Salema NE, et al. Supporting adherence for people starting a new medication for a long-term condition through community pharmacies: A pragmatic randomised controlled trial of the New Medicine Service. *BMJ quality & safety* 2016;25(10):747-58.
10. Latif A, Pollock K, Boardman HF. The contribution of the Medicines Use Review (MUR) consultation to counseling practice in community pharmacies. *Patient Educ Couns* 2011;83(3):336-44.
11. Gann B. Understanding and using health experiences: the policy landscape. In: Ziebland S, Coulter A, Calabrese JD, et al., eds. Understanding and using health experiences: improving patient care. Oxford, United Kingdom: Oxford University Press 2013.
12. Gidman W, Ward P, McGregor L. Understanding public trust in services provided by community pharmacists relative to those provided by general practitioners: a qualitative study. *BMJ Open* 2012;2(3)
13. Hall MA, Dugan E, Zheng B, et al. Trust in physicians and medical institutions: what is it, can it be measured, and does it matter? *Milbank Q* 2001;79(4):613-39, v. [published Online First: 2002/01/16]
14. Latif A, Boardman HF, Pollock K. Understanding the patient perspective of the English community pharmacy Medicines Use Review (MUR). *Res Social Adm Pharm* 2013;9(6):949-57.

For peer review only

For peer review only - http://bmjopen.bmj.com/site/about/guidelines.xhtml

15. Tinelli M, Ryan M, Bond C. Patients' preferences for an increased pharmacist role in the management of drug therapy. *Int J Pharm Pract* 2009;17(5):275-82.
16. Latif A, Waring J, Watmough D, et al. Examination of England's New Medicine Service (NMS) of complex health care interventions in community pharmacy. *Res Social Adm Pharm* 2016;12(6):966-89.
17. Bereznicki B, Peterson G, Jackson S, et al. Perceived feasibility of a community pharmacy-based asthma intervention: a qualitative follow-up study. *J Clin Pharm Ther* 2011;36(3):348-55.
18. Bradley F, Wagner AC, Elvey R, et al. Determinants of the uptake of medicines use reviews (MURs) by community pharmacies in England: a multi-method study. *Health Policy* 2008;88(2):258-68.
19. Naik Panvelkar P, Armour C, Saini B. Community pharmacy-based asthma services-what do patients prefer? *J Asthma* 2010;47(10):1085-93.
20. Braun V, Clarke V. Using thematic analysis in psychology. *Qualitative Research in Psychology* 2006;3(2):77-101.
21. Sturges JE, Hanrahan KJ. Comparing Telephone and Face-to-Face Qualitative Interviewing: a Research Note. *Qualitative Research* 2004;4(1):107-18.
22. Boyd MJ, Elliott R, Barber N, et al. The impact of the New Medicines Service (NMS) in England on patients adherence to their medicines. *Int J Pharm Pract* 2014;22:66.
23. British Thoracic Society/Scottish Intercollegiate Guidelines Network. British guideline on the management of asthma: a national clinical guideline. Edinburgh: British Thoracic Society/Scottish Intercollegiate Guidelines Network, 2016:1-172.
24. Centre for Pharmacy Postgraduate Education. Clinical pharmacists in general practice education. Manchester, United Kingdom: University of Manchester, 2018.
25. Horne R, Weinman J. Self-regulation and self-management in asthma: exploring the role of illness perceptions and treatment beliefs in explaining non-adherence to preventer medication. *Psychol Health* 2002;17(1):17-32.

For peer review only

For peer review only - http://bmjopen.bmj.com/site/about/guidelines.xhtml

APPENDIX A: DESCRIPTION OF A PHARMACIST-LED ADHERENCE CONSULTATION DELIVERED IN GENERAL PRACTICE

We will describe this service as if you were receiving it.

The service

The service is delivered by a clinical pharmacist based at your local GP practice. The aim is to **help people with asthma get on better with their inhalers**. This means answering all of your questions and discussing all of your concerns about your inhalers in detail.

Pre-consultation

You are booked in for a consultation with the clinical pharmacist by the GP receptionist (just like you would with a GP appointment).

The main consultation

You have a one-to-one consultation with the pharmacist in a consultation room. They will begin with a **standard asthma review**. You will talk about your asthma control, recent symptoms, smoking history, and your asthma action plan. They will check your inhaler technique and lung function.

You will then have an **in-depth discussion about your inhalers**. You can ask any questions or mention any concerns you have about your inhalers. This could be anything from concerns about side effects or questions about how the medication works. The pharmacist will give you useful information and feedback that is **specifically suited to you**. They may use a short video or print-outs to guide their discussion with you.

At the end of the consultation, you will set an asthma-related goal with the pharmacist. This could be something like "less night time asthma symptoms". The pharmacist will advise you on how to achieve your personal goal using your inhalers.

The follow-up consultation

1 month later, you will see the pharmacist again for a short consultation (no more than 10 minutes). The pharmacist will briefly ask how your asthma has been and check your inhaler technique/lung function. You will then discuss how you are getting on with your medicines and your personal asthma goal. Based on this discussion, the pharmacist will give further recommendations regarding your medication.

For peer review only - http://bmjopen.bmj.com/site/about/guidelines.xhtml

APPENDIX B: INTERVIEW TOPIC GUIDE

Background and Previous Experience

1. To start the interview, could you tell me when you were diagnosed with asthma?

2. What type of medications are you currently prescribed for your asthma?

    a. How has your experience been with these medications? (Prompting to see if there are concerns, side effects etc.)

3. A lot of people with asthma have questions or concerns about their inhalers or tablets. What do you do when you have any questions or concerns specifically about your asthma medication?

    a. Prompts: do you do research online? Do you talk to friends and family? Do you go talk to a healthcare professional?
    b. Could you tell me a bit about why you prefer (insert route, e.g. researching online, talking to the GP, talking to family etc.) when it comes to your medication?

4. Have you ever talked to a pharmacist about your asthma medication?

    a. If **YES**: Could you walk me through what happened during that interaction?
        i. Where did it take place? (community pharmacist, hospital pharmacist etc.)
        ii. Prompts: what type of information did they give you? Were they able to answer your questions? Did you feel comfortable talking to them about your asthma medication? Did you feel like it was helpful for you?
        iii. Would you consider the pharmacist as a regular part of your asthma care team? Why or why not?

    b. If **NO**: Could you imagine a pharmacist as part of your regular asthma care team?

5. I'd now like to talk specifically about your preventer inhaler (brown inhaler, steroid inhaler). They're normally prescribed for daily or twice daily use. A lot of asthma patients we've talked to often don't take the inhaler as regularly as prescribed, is this something you've experienced as well?
    a. Could you tell me a little bit about why?

Pharmacist in general practice

Great, that's the first part of our interview. Now we're going to continue on to the part where I'd like to get your thoughts on pharmacist-led consultations in general practice. I've e-mailed you a description of the service already, but would you like to review it to refresh your memory?

For peer review only - http://bmjopen.bmj.com/site/about/guidelines.xhtml

Do you have any questions or anything you would like me to clarify before we move on to the questions?

6. What are your initial thoughts on this pharmacist medication support service?
    a. Explore which aspects of the service are most salient to the participant, use prompts below when needed.

- In your opinion, would this type of service be a useful addition to the care you get from your GP/nurse/asthma specialist?
    - Is this a type of service you could see yourself using? Why or why not?
    - Do you think this service is any different from the asthma care you already receive?
- Do you think this type of service would help you take your preventer inhaler more regularly?
- How do you feel about a pharmacist taking on this type of clinical role?
    - Do you think pharmacists have enough training for this type of work?
        - What type of knowledge would you expect the pharmacist to have?
    - How do pharmacists compare to your other healthcare professionals (e.g. GP or nurse)?
    - Do you think this service is any different from seeing a community pharmacist? (Are they familiar with the NMS and MUR?)
    - Would you feel comfortable talking to a pharmacist about your asthma and your medication?
    - Do you feel like you can trust a pharmacist in this type of role?
- How do you feel about having a pharmacist based in a GP surgery?
    - How would you feel about this pharmacist having access to your medical records?
- What do you think about seeing a pharmacist on an appointment basis?
- When you think about (concerns/side effects discussed for question 2a), do you think those types of questions or concerns could be addressed by a pharmacist?
    - What would be the ideal way to support you with those questions or concerns?
- Do you think this type of service is convenient for people with asthma?

For peer review only

Reporting checklist for qualitative study.

Based on the SRQR guidelines.

Instructions to authors

Complete this checklist by entering the page numbers from your manuscript where readers will find each of the items listed below.

Your article may not currently address all the items on the checklist. Please modify your text to include the missing information. If you are certain that an item does not apply, please write "n/a" and provide a short explanation.

Upload your completed checklist as an extra file when you submit to a journal.

In your methods section, say that you used the SRQRreporting guidelines, and cite them as:

O'Brien BC, Harris IB, Beckman TJ, Reed DA, Cook DA. Standards for reporting qualitative research: a synthesis of recommendations. Acad Med. 2014;89(9):1245-1251.

		Reporting Item	Page Number
**Title**			
	#1	Concise description of the nature and topic of the study identifying the study as qualitative or indicating the approach (e.g. ethnography, grounded theory) or data collection methods (e.g. interview, focus group) is recommended	0
**Abstract**			
	#2	Summary of the key elements of the study using the abstract format of the intended publication; typically includes background, purpose, methods, results and conclusions	1
**Introduction**			
Problem formulation	#3	Description and signifcance of the problem / phenomenon studied: review of relevant theory and empirical work; problem statement	2-3
Purpose or research question	#4	Purpose of the study and specific objectives or questions	3

For peer review only

Methods

Qualitative approach and research paradigm	#5	Qualitative approach (e.g. ethnography, grounded theory, case study, phenomenolgy, narrative research) and guiding theory if appropriate; identifying the research paradigm (e.g. postpositivist, constructivist / interpretivist) is also recommended; rationale. The rationale should briefly discuss the justification for choosing that theory, approach, method or technique rather than other options available; the assumptions and limitations implicit in those choices and how those choices influence study conclusions and transferability. As appropriate the rationale for several items might be discussed together.	3
Researcher characteristics and reflexivity	#6	Researchers' characteristics that may influence the research, including personal attributes, qualifications / experience, relationship with participants, assumptions and / or presuppositions; potential or actual interaction between researchers' characteristics and the research questions, approach, methods, results and / or transferability	5
Context	#7	Setting / site and salient contextual factors; rationale	5
Sampling strategy	#8	How and why research participants, documents, or events were selected; criteria for deciding when no further sampling was necessary (e.g. sampling saturation); rationale	3-5
Ethical issues pertaining to human subjects	#9	Documentation of approval by an appropriate ethics review board and participant consent, or explanation for lack thereof; other confidentiality and data security issues	17
Data collection methods	#10	Types of data collected; details of data collection procedures including (as appropriate) start and stop dates of data collection and analysis, iterative process, triangulation of sources / methods, and modification of procedures in response to evolving study findings; rationale	5
Data collection instruments and technologies	#11	Description of instruments (e.g. interview guides, questionnaires) and devices (e.g. audio recorders) used for data collection; if / how the instruments(s) changed over the course of the study	5
Units of study	#12	Number and relevant characteristics of participants, documents, or events included in the study; level of participation (could be	6

For peer review only

		reported in results)	
Data processing	#13	Methods for processing data prior to and during analysis, including transcription, data entry, data management and security, verification of data integrity, data coding, and anonymisation / deidentification of excerpts	5
Data analysis	#14	Process by which inferences, themes, etc. were identified and developed, including the researchers involved in data analysis; usually references a specific paradigm or approach; rationale	5-6
Techniques to enhance trustworthiness	#15	Techniques to enhance trustworthiness and credibility of data analysis (e.g. member checking, audit trail, triangulation); rationale	6
**Results/findings**			
Syntheses and interpretation	#16	Main findings (e.g. interpretations, inferences, and themes); might include development of a theory or model, or integration with prior research or theory	7-13
Links to empirical data	#17	Evidence (e.g. quotes, field notes, text excerpts, photographs) to substantiate analytic findings	7-13
**Discussion**			
Intergration with prior work, implications, transferability and contribution(s) to the field	#18	Short summary of main findings; explanation of how findings and conclusions connect to, support, elaborate on, or challenge conclusions of earlier scholarship; discussion of scope of application / generalizability; identification of unique contributions(s) to scholarship in a discipline or field	13-16
Limitations	#19	Trustworthiness and limitations of findings	13-14
**Other**			
Conflicts of interest	#20	Potential sources of influence of perceived influence on study conduct and conclusions; how these were managed	17
Funding	#21	Sources of funding and other support; role of funders in data collection, interpretation and reporting	17

The SRQR checklist is distributed with permission of Wolters Kluwer © 2014 by the Association of American Medical Colleges. This checklist was completed on 01. June 2019 using https://www.goodreports.org/, a tool made by the EQUATOR Network in collaboration with Penelope.ai

For peer review only

Pharmacist-led adherence support in general practice: a qualitative interview study of adults with asthma

Journal:	*BMJ Open*
Manuscript ID	bmjopen-2019-032084.R1
Article Type:	Original research
Date Submitted by the Author:	30-Aug-2019
Complete List of Authors:	Mes, Marissa; UCL School of Pharmacy, Centre for Behavioural Medicine, Practice & Policy<br>Katzer, Caroline; UCL School of Pharmacy, Centre for Behavioural Medicine, Practice & Policy<br>Wileman, Vari; UCL School of Pharmacy, Centre for Behavioural Medicine, Practice & Policy<br>Chan, Amy; UCL School of Pharmacy, Centre for Behavioural Medicine, Practice & Policy<br>Horne, Robert; UCL School of Pharmacy, Centre for Behavioural Medicine, Practice & Policy<br>Taylor, Stephanie; Queen Mary University of London, Centre for Primary Care and Public Health
**Primary Subject Heading**:	General practice / Family practice
Secondary Subject Heading:	Respiratory medicine, Health services research
Keywords:	Medication Adherence, Asthma < THORACIC MEDICINE, Pharmaceutical Services, General Practice, Medication Therapy Management

SCHOLARONE™
Manuscripts

Pharmacist-led adherence support in general practice: a qualitative interview study of adults with asthma

Mes, Marissa Ayano[1]; Katzer, Caroline Brigitte[1]; Wileman, Vari[1]; Chan, Amy Hai Yan[1]; Horne, Rob[1]; & Taylor, Stephanie Jane Caroline[2].

[1]Centre for Behavioural Medicine, UCL School of Pharmacy, Mezzanine Floor, Tavistock Square, London WC1H 9JP, London, United Kingdom.

[2]Centre for Primary Care and Public Health, Blizard Institute, Queen Mary University London, Yvonne Carter Building, 58 Turner Street, London E1 2AB, United Kingdom.

Corresponding author: Marissa Ayano Mes (marissa.mes.15@ucl.ac.uk)

Word count: 3,936 words

For peer review only

Abstract

Objectives: The National Health Service (NHS) in England recently introduced general practice pharmacists (GPPs) to provide medication-focused support to both patients and the general practice team. This healthcare model may benefit people with asthma, who currently receive sub-optimal care and demonstrate low medication adherence. This study aimed to explore the perspectives of adults with asthma on the potential for pharmacist-led adherence support delivered in general practice, with a focus on how these perspectives are formed.

Design and setting: The study was conducted in the United Kingdom (UK) utilising a qualitative interview methodology. Participants were invited to partake in a telephone-based semi-structured interview, followed by an online questionnaire for demographic details and asthma history. Qualitative data was analysed using thematic analysis.

Participants: Participants (n = 17) were adults with asthma in the UK with a prescription for an inhaled corticosteroid. Participants did not have previous experience with GPPs and were asked to provide their views on a proposed GPP-led service.

Results: Participant perspectives of GPPs were determined by trust in pharmacists, perceived gaps in asthma care, and the perceived strain on the NHS. Trust was based on pharmacists' perceived clinical competency, established over time, and gauged through a 'benchmarking' process. GPP's fit in current asthma care was assessed based on potential role overlap with other healthcare professionals, continuity of care, and medication-related support needs. Participants navigated the NHS based on a perceived hierarchy of healthcare professionals (GPs on top, nurses, then pharmacists), and this influenced their perspectives of GPPs.

For peer review only

Conclusions: While the GPP scheme shows promise based on the perspectives of people with asthma, the identified barriers to optimal patient engagement and service implementation will need to be addressed for the service to be effective.

Strengths and limitations of this study

- The use of qualitative methodology captured the complex processes behind people's perceptions of general practice pharmacists (GPPs), including how lived experiences shaped perceptions of new pharmacist-led services.
- Telephone-based interviews enabled recruitment across the United Kingdom (UK) and increased study accessibility for people with severe asthma and travel limitations.
- The study may not have captured the full variation in views among adults with asthma in the UK because participants primarily had self-reported mild asthma and were recruited through an asthma charity.
- Participants had no experience with GPP-led consultations and therefore represented the general population with asthma that would initially need to be convinced to engage with the service.

For peer review only

Background

The pressure on primary care to deliver core services is increasing rapidly worldwide due to a growing and ageing population, the prevalence of long-term conditions, and significant resource constraints. Primary care systems are being reshaped and new models of care are emerging to cope with growing demand.[1]

One such model is the general practice pharmacist (GPP) model, which was introduced in England as part of the NHS *General Practice Forward View* initiative.[2-4] These pharmacists

For peer review only - http://bmjopen.bmj.com/site/about/guidelines.xhtml

support both patients and the general practice team with medication-related issues, with the aim of expanding the general practice workforce, reducing practice burden, and increasing patient access to appointments.[4] Initial qualitative feedback from general practitioners (GPs) and pharmacists in a pilot study in England suggests that GPPs can have positive impacts on medication safety, medication adherence, healthcare access, and patient satisfaction across a variety of long-term conditions.[3]

People with asthma may benefit from GPP support. Research shows that adherence to inhaled corticosteroids (ICS), an essential medication for asthma, is low.[5,6] Furthermore, only 35% of people with asthma in the United Kingdom (UK) receive basic care (i.e. annual reviews, inhaler technique checks, and a written asthma action plan).[7] Previous research suggests that pharmacist-led interventions can increase medication adherence and support asthma self-management among people with asthma.[8] However, most of the previous research in the UK has been limited to community rather than general practice pharmacists, and focused on their impact across a range of long-term conditions rather than asthma specifically.[9,10]

Understanding the specific perspectives of people with asthma regarding GPPs is an important first step in establishing the potential benefits of this new service for this specific patient group, as well as identifying any potential issues in its future uptake and effectiveness.[11] The aim of this study was to explore the perspectives of adults with asthma on the potential of pharmacist-led asthma adherence support delivered in general practice, with a focus on how these perspectives are formed.

Methods

This study is reported according to the Standards for Reporting Qualitative Research.[12] It was a telephone-based semi-structured interview study, using an interpretivist approach to

For peer review only - http://bmjopen.bmj.com/site/about/guidelines.xhtml

understand how adults with asthma construct their initial opinions of GPP-led asthma adherence support. Demographic details and asthma history were collected using a brief online questionnaire.

Participants

Participants were adults (≥ 18 years old) living in the UK and proficient in English, with a self-reported asthma diagnosis, a prescription for ICS, and access to a telephone and e-mail account. People with respiratory comorbidities (e.g. Chronic Obstructive Pulmonary Disease) and/or those in hospital or nursing homes were excluded, as the adherence behaviour and support needs of these individuals were hypothesised to be different.

Recruitment

Several recruitment channels were used to ensure that participants varied in age, gender, and self-reported asthma severity. A flyer with study information and researchers' contact details was circulated by researchers, the Asthma UK Centre for Applied Research (AUKCAR), and the National Institute for Health Research Collaboration for Leadership in Applied Health Research and Care North Thames via social media. The study was advertised in two electronic newsletters: the Asthma UK volunteer bulletin and the University College London student newsletter. Printed flyers were handed directly to potential participants by a respiratory consultant at a London hospital, and pharmacists at two hospitals in Wales.

People who contacted the researchers were e-mailed an information sheet, consent form, and eligibility criteria to review. If they were eligible and willing to participate, they were booked in for a one-hour telephone interview. In preparation for the call, participants were asked to read a description of a GPP-led adherence support consultation, sent to them via e-mail (see Appendix A). This description was based on the work of a clinical respiratory pharmacist

For peer review only - http://bmjopen.bmj.com/site/about/guidelines.xhtml

working in general practice in London. Participants gave verbal consent over the telephone before the interview began. The consent procedure and interview were audio-recorded with their permission. All participants received a £20 online shopping voucher to thank them for their time.

Data collection and analysis

One researcher (MM) conducted the interviews. Participants were informed that the researcher had a background in Health Psychology and an interest in adherence and pharmacist-led care for asthma.

The interview topic guide had two sections (see Appendix B). The first section focused on participants' previous experiences of asthma, asthma care, and pharmacists. The second section focused on how these lived experiences informed participants' opinions of GPP-led adherence support, with questions based on previous research on interpersonal/institutional trust in healthcare professionals,[13 14] perceptions of the pharmacist role,[15-17] and pharmacist-led care for asthma.[10 18-20]

Participants also completed an online questionnaire on demographic details (gender and age) and asthma history (self-reported asthma severity, hospitalisations, and GP visits). An online questionnaire was used because participants may have felt uncomfortable disclosing personal information (e.g. age) directly to a researcher during the telephone call. The self-report method was chosen because recruitment to the study may have been difficult if access to participants' medical records was required.

All interviews were professionally transcribed, with transcripts checked for accuracy by MM. Data were analysed using NVivo (QSR, Version 11). Thematic analysis was used to identify themes at the semantic level using a deductive approach, based on their relevance to the study

For peer review only - http://bmjopen.bmj.com/site/about/guidelines.xhtml

aim.[21] Continuous iterative analyses were conducted to establish when thematic saturation had been reached. Recruitment was set for 30 participants or thematic saturation, whichever was attained first. The study ran from October 2017 to February 2018. All transcripts were analysed by MM, and four transcripts (representing 25% of the total transcripts) were independently second-coded by another researcher (CK), as recommended by MacPhail, et al. [22]. Discrepancies were resolved through consensus discussion.

Patient and public involvement

All study materials (recruitment flyer, participant information sheet, consent form, interview topic guide, GPP consultation example, and online questionnaire) were reviewed by the AUKCAR Patient Advisory Group (PAG) prior to study commencement. Members of the PAG were adults with asthma. Their feedback, often regarding word choice and text length, was incorporated into the study materials.

Results

Thematic saturation was reached with 17 participants (Table 1). The mean interview length was 39 minutes (ranging from 30 to 58 minutes). The participant sample was 59% female, with most participants (41%) aged 30 to 39 years and recruited through the Asthma UK newsletter (41%). The sample included participants with self-reported mild (53%), moderate (24%), and severe asthma (17%). Three overarching themes (with seven sub-themes) were identified from the data: building trust in pharmacists, filling gaps in current asthma care, and navigating a strained healthcare system.

Table 1. Demographic characteristics and asthma history

Characteristics (n = 17)	Frequency n (%)
**Gender**	

For peer review only - http://bmjopen.bmj.com/site/about/guidelines.xhtml

Female	10 (59%)
Male	7 (41%)
**Age in years**	
18 – 29	5 (29%)
30 – 39	7 (41%)
40 – 49	2 (12%)
50 - 59	-
60 – 69	2 (12%)
70 +	1 (6%)
**Recruitment channel**	
Asthma UK newsletter	7 (41%)
Hospital	1 (6%)
Social media	3 (17%)
University College London newsletter	3 (18%)
Word of Mouth	3 (18%)
**Self-reported asthma severity**	
Mild	9 (53%)
Moderate	4 (24%)
Severe	3 (17%)
Prefer not to disclose	1 (6%)
**Self-reported hospitalisations for asthma (previous 12 months)**	
0	11 (65%)
1 – 4	3 (17%)
5 – 10	3 (17%)
**Self-reported GP visits for asthma (previous 12 months)**	
0	2 (12%)
1 – 10	13 (76%)
10 – 20	2 (12%)

Theme 1: building trust in pharmacists

Trust in healthcare professionals involves the optimistic acceptance of being in a vulnerable situation, knowing that one's interests will be cared for.[14] For participants, opinions of the new service were based on the level of trust they placed in pharmacists. Trust was built over time, based on perceived clinical competency, and through a benchmarking process, which form the sub-themes discussed below.

Building trust over time

Participants highlighted that trust in any healthcare professional builds through consistent contact over time. Some participants felt hesitant about the new service because it meant

For peer review only - http://bmjopen.bmj.com/site/about/guidelines.xhtml

deviating from their usual trusted healthcare professional, suggesting a preference for usual care over new initiatives to maintain the quality of their asthma care.

"I don't really know, I think I'd prefer a doctor [to talk to about my asthma]. It's the way it's always been."

– P15, male, 30 – 39 years, mild asthma

Building trust based on perceived clinical competency

When asked about specific criteria for trust, participants discussed pharmacists' clinical competency. This included pharmacists' asthma-specific and broad clinical knowledge. Support for the new service was high when pharmacists were viewed as knowledgeable.

"So I know that in the pharmacy role they're very knowledgeable. So if [adherence support for asthma] is something they want to do then why not? I have a lot of faith in somebody who's got a lot of knowledge in something."

– P8, female, 30 – 39 years, severe asthma

Some participants believed that pharmacists would need extensive additional training to develop the knowledge needed for the new service. Their main concern was that pharmacists were too medication-focused and therefore lacked broader clinical skills.

"...it could be that the [medication] side-effects are something else entirely. So [pharmacists] would be kind of completely thinking down the asthma route, 'it might just be that you're taking an inhaler that you feel side-effects'...but what if it turns out you actually have cancer?"

– P17, male, 18 – 29 years, mild asthma

Building trust through a benchmarking process

None of the participants had previous experiences with GPPs, with some participants questioning the differences between GPPs and community pharmacists. Many participants engaged in a *benchmarking* process: using their trust and previous experiences of other healthcare professionals to gauge how much they could trust a GPP. Common reference

For peer review only - http://bmjopen.bmj.com/site/about/guidelines.xhtml

points included community pharmacists, respiratory consultants, nurses, GPs, and paramedics.

"…I know [pharmacist support] is there but I still don't understand it with [brittle] asthma because I still get wary. If paramedics have never heard of it and don't know what they're doing, how's a pharmacist going to hear of it?"
– P2, female, 30 – 39 years, severe asthma

"I would much rather go to a pharmacist than to a nurse to discuss the medication issues that I was having… I can see an asthma nurse to discuss medication, and I was like 'Really? What do nurses know about…not to be rude, but what do they know about medication more than my specialist who prescribed it?"
– P4, female, 30 – 39 years, mild asthma

Theme 2: filling gaps in current asthma care

Participants' opinions of GPPs were also informed by perceived gaps in their current asthma care. Participants evaluated the new service's place in their current care based on potential role overlap between GPPs and other healthcare professionals, continuity of care, and medication-specific support.

Potential role overlap

Participants that saw potential role overlap between GPPs and other healthcare professionals were more sceptical of the new service.

"I think if I was having an annual asthma review I wouldn't need to use the pharmacist's service as well, but it might be an alternative to the annual asthma review…"
– P3, male, 70+ years, mild asthma

However, other participants clearly delineated the GPP role, and these participants often recommended ways to integrate pharmacists into their care.

"If you're asking a GP, you've got maybe five, ten minutes… If you know you've got another ten or fifteen minutes with this pharmacist… for asking all these questions…with the GP you can concentrate on the problem and get that sorted, and then go see the pharmacist and discuss the medication."
– P5, female, 60 – 69 years, moderate asthma

For peer review only - http://bmjopen.bmj.com/site/about/guidelines.xhtml

Continuity of care

Participants with self-reported severe asthma often had multiple healthcare professionals involved in their care (e.g. respiratory consultants, GPs, and asthma nurses). When asked about the new service, some participants felt concerned about involving an additional healthcare professional in their care. This was unrelated to their views on pharmacist competency, and was usually influenced by previous experiences of inadequate continuity of care due to a lack of communication between healthcare professionals.

"[Pharmacists] always say speak to your GP but then the GP tells you to speak to the pharmacist because they're supposed to know more about drugs than what they are…and then you're somewhere in the middle…"
– P2, female, 30 – 39 years, severe asthma

Medication-specific support

Other participants with severe asthma on multiple medications and/or with other health concerns welcomed the service. This enthusiasm came from the fact that they identified gaps in their current care that they believed could be filled by pharmacists as medication experts.

"…just having contact with someone who actually…knows about the medication, like they know how they work and what the potential side effects are going to be and interactions…it's that knowledge that a GP wouldn't necessarily have time to tell you all about…"
– P6, female, 30 – 39 years, severe asthma

"…I'm trying to conceive at the moment so…and I thought I don't want to be taking anything that's unnatural or steroid-y…I did ask the respiratory consultant [about asthma medications and In Vitro Fertilisation] but he didn't know…"
–P8, female, 30 – 39 years, severe asthma

Some participants felt that GPPs should have an independent prescribing qualification to fulfil their role as medication experts. They worried that the new service might contribute to the burden on patients and/or the healthcare system, and that independent pharmacist prescribers would minimise the risk of this happening.

For peer review only - http://bmjopen.bmj.com/site/about/guidelines.xhtml

"For me, it would just be down to whether or not [pharmacists] are able to prescribe. I don't imagine that they wouldn't have the knowledge that was required…It's just if I had to then see a doctor to be prescribed a different medication, I'd rather just go to see the doctor instead."

– P10, female, 40 – 49 years, mild asthma

Theme 3: navigating a strained healthcare system

Participants were acutely aware of the limited resources within general practice. They often expressed guilt and frustration about booking appointments for asthma. Participants never booked appointments just for medication-related questions, and their medication-related concerns were frequently left unaddressed because other topics took priority in a consultation, particularly if the participant had multiple comorbidities. The pharmacist-led service was welcomed by these participants because they felt pharmacists would have more time to focus on their medication.

"…come Monday morning I wouldn't want to call the GP because I know on Monday morning they're very, very busy…I'll just sort of crack on at home, multi-dosing salbutamol and seeing what happens."

– P8, female, 30 – 39 years, severe asthma

"[GPs] just want you in and out… 'oh yes, I wanted to ask you something else' but too late now, you're away. That's how you feel."

–P1, female, 30 – 39 years, mild asthma

The hierarchy of healthcare professionals

Many participants constructed a hierarchy of healthcare professionals with GPs at the top, followed by nurses and finally pharmacists. This hierarchy determined the importance of each healthcare professional's time. Severe health concerns justified booking a GP appointment, while non-urgent concerns were viewed as more suitable for pharmacists.

The hierarchy of healthcare professionals affected perceptions of GPPs in both directions. Some participants were enthusiastic about the new service because they believed it would

For peer review only - http://bmjopen.bmj.com/site/about/guidelines.xhtml

lessen the workload of GPs and nurses. For these participants, seeing a pharmacist (the healthcare professional further down the hierarchy) felt less intimidating and formal, slightly easing concerns about taking up valuable appointment time.

"…to be honest, GPs have bigger problems to deal with…[they're] dealing with people with, you know, life threatening illnesses, then actually seeing the standard case of asthma or an asthma check-up isn't the best use of [their] time."
– P16, male, 18 – 29 years, moderate asthma

"It feels less formal, I think, when you're with a pharmacist than when you're in the doctor's…sometimes when you go to the doctor's, you're kind of clock watching…"
– P10, female, 40 – 49 years, mild asthma

Others felt that pharmacists could not extend into a clinical role similar to GPs and nurses, with some suggesting a triage-like function to safeguard GP time.

"I never feel as though a pharmacist is a nurse, if you see what I mean. A nurse has practical hands-on experience of trying to make people better. The pharmacist is one who deals with the theory of medication."
–P9, male, 60 – 69 years, undisclosed asthma severity

"… the pharmacist has seen you and if there's communication between the pharmacist and the GP, so that I guess it would help the GPs prioritise who they saw…"
–P11, female, 40 – 49 years, moderate asthma

However, pharmacists themselves were also viewed as a limited resource. Many participants supported moving pharmacists from community pharmacies to general practice because they experienced inadequate care in busy community pharmacies. Others were concerned that pharmacist-led adherence support with a wide scope (i.e. for multiple long-term conditions) would limit access for people with asthma.

"If [pharmacists] weren't running a community pharmacy, if they were linked in, if they worked within the GP surgery with a lot of time, then yes, I don't see how [a lack of time] would be an issue."
– P12, female, 30 – 39 years, mild asthma

"…my worry is if a pharmacist has to do [adherence support in general practice] for asthma, what other long-term conditions will they have to do it for?"
– P6, female, 30 – 39 years, severe asthma

For peer review only - http://bmjopen.bmj.com/site/about/guidelines.xhtml

Discussion

This is the first in-depth exploration of the perspectives of adults with asthma on pharmacist-led adherence support in general practice. This focused exploration identified potential barriers to service uptake, and has the potential to help further refine and tailor the GPP service as it is rolled out.

Interpretation of findings

Trust played an important role in participants' initial perspectives of GPP-led care – it was an essential component of the patient-pharmacist relationship and it informed participants' views of pharmacists' role in asthma care. These findings suggest that general awareness of the existence of pharmacist-led services is insufficient to encourage service uptake.

Participants based their trust in pharmacists on perceived clinical competency and comparisons with other trusted healthcare professionals (e.g. GPs and nurses), guided by perceptions of pharmacists' position in the hierarchy of healthcare professionals. These findings may suggest that explicit endorsement of GPP-led care by other trusted healthcare professionals might improve service uptake among adults with asthma. Reassurance and support from GPs and nurses may address some of the concerns raised by study participants, including those about fragmented care, pharmacists' clinical competency, and potential role overlap.

Support for GPP-led care did not seem to differ based on participants' age, gender, or self-reported asthma severity – there was a variety of views in each group of participants. However, participants on multiple medications or those with additional health concerns seemed to be more open to pharmacist input, perhaps because they felt current asthma care

For peer review only - http://bmjopen.bmj.com/site/about/guidelines.xhtml

was unable to meet these additional needs. Targeting this group of adults with asthma may improve service uptake in the future.

Strengths and limitations

The qualitative method in this study captured the complex processes behind people's initial opinions of GPPs. The combination of recruitment channels produced variation in the sample in terms of age, self-reported asthma severity, and healthcare utilisation. Telephone-based interviews enabled recruitment across the UK without increasing participant burden, thus increasing study accessibility for participants with severe asthma and limited travel capacity. In addition, telephone-based interviews can produce data of higher quality compared to face-to-face interviews when sensitive topics (e.g. long-term conditions) are discussed.[23]

The participant sample may however not have captured the views of all adults with asthma because it consisted primarily of people with self-reported mild asthma (53%) and people recruited through Asthma UK (41%). Thematic saturation may have been reached due to the relative homogeneity of the participant sample. The participants recruited through Asthma UK may have had a stronger interest in asthma care or pharmacist-led support, and may therefore have been more supportive of the new service compared to the general population with asthma. However, if scepticism of the new service exists among people who are more engaged in their care, then the findings may be amplified in the general population with asthma who may have less interest in asthma care.

A major drawback of the study is that none of the participants had experienced a GPP consultation directly, with some participants recruited from Scotland and Northern Ireland where the GPP scheme does not exist. While the consultation description that participants were asked to read was based on real work by a clinical respiratory pharmacist working in general practice, study findings can only be used to understand patients' initial views of a

For peer review only - http://bmjopen.bmj.com/site/about/guidelines.xhtml

GPP-led service. These participants represent the general population with asthma who would initially need to be convinced to engage with the service. Addressing some of the concerns identified by participants may help improve the uptake of existing GPP-led care among adults with asthma. However, participants' views may change over time, and could potentially be influenced by direct personal experience with a GPP consultation.

Comparison with existing literature

There is limited research on patient perspectives of GPPs because the care model is relatively new. However, findings from this study align with those from community pharmacy-based research, suggesting that people with asthma may not differentiate between pharmacy sectors.

Findings align with work by Gidman, et al. [13], who found that people were hesitant about deviating from their usual trusted care model (often a GP). However, a few of the older participants in this study were open to GPP-led care, in contrast to previous research that suggests that older patients may be less likely to accept an expanded pharmacist role.[24]

Both general members of the public and people with asthma have been found to use other trusted healthcare professionals (e.g. GPs and nurses) as benchmarks for trust when asked about a community pharmacist-led service.[13 20] Similarly, Naik Panvelkar, et al. [20] found that previous positive experiences with community pharmacists raised expectations for other pharmacist-led services in a population of people with asthma.

Participants' views of the gaps in their current asthma care shaped their perspectives of GPPs. Similarly, Boyd, et al. [25] found that recipients of the New Medicine Service (NMS) welcomed pharmacists' recommendations if they addressed a concern directly raised by the patient. While asthma care guidelines recommend a multidisciplinary approach in treating difficult asthma, the present study suggests that some people with self-reported severe asthma

For peer review only - http://bmjopen.bmj.com/site/about/guidelines.xhtml

were hesitant to include another healthcare professional due to issues with continuity of care.[26] In line with previous research, the hierarchy of healthcare professionals influenced perspectives of pharmacists expanding further into clinical roles.[13 15] However, this study also found that the hierarchy increased support for pharmacist-led care to reduce the burden on GPs/nurses.

Implications for research and practice

As the GPP model is rolled out, future studies could be conducted using in-depth interviews with people with asthma after they have experienced a GPP-led consultation. These interviews could establish if interpersonal factors (i.e. rapport with the pharmacist) have an impact on patient perspectives. Ethnographic observations of pharmacist-led consultations and the general practice team will help assess pharmacists' integration and its effect on continuity of care for asthma patients. Future recruitment should aim for greater variation in participants (e.g. self-reported asthma severity) through various recruitment channels, with additional efforts to look for discordant voices when thematic saturation is reached.

Findings from this study could be implemented in efforts to increase service uptake among people with asthma. Given the *benchmarking* process used to establish trust in pharmacists, comparisons between GPPs and other healthcare professionals could be used to inform and engage the public. For example, public campaigns highlighting the differences and similarities between GPs and GPPs may help the public differentiate the pharmacist role and understand the added value of the new service within asthma care.

Participants wanted GPPs to have broad clinical skills and a prescribing qualification. Encouragingly, the Centre for Pharmacy Postgraduate Education has already included these components in their GPP training pathways.[27]

For peer review only - http://bmjopen.bmj.com/site/about/guidelines.xhtml

Although the hierarchy of healthcare professionals sometimes prevented pharmacists from being perceived as clinicians, it also made GPP appointments appear less formal and intimidating to access. Participants felt more comfortable making an appointment with a pharmacist for medication-related questions. This is encouraging because the new service may encourage people with asthma to address medication-related concerns that may be barriers to medication adherence.[28]

While the perspectives of people with asthma explored in this study show that the GPP model has promise, they identified several barriers to optimal patient engagement and service implementation that will need to be addressed for the service to be effective. Meeting patient expectations will be the first crucial step in ensuring the programme's long-term benefit and reducing the pressure on general practice in England.

Acknowledgements

We would like to thank the Asthma UK Centre for Applied Research (AUKCAR) Patient Advisory Group for their feedback and support for this study.

Contributors

The study was designed by MM, RH, and SJCT. All interviews were conducted by MM. Data was analysed by MM and CK. The manuscript was written by MM, AHYC, and VW, with input from RH and SJCT.

Funding

The research was funded by the National Institute for Health Research (NIHR) Collaboration for Leadership in Applied Health Research and Care North Thames at Barts Health NHS

For peer review only

Trust. The views expressed are those of the authors and not necessarily those of the NHS, the NIHR, or the Department of Health and Social Care.

Competing interests

None declared.

Patient consent for publication

Not required.

Ethics approval

The research was approved by the NHS London-Harrow Research Ethics Committee (12th October 2017, Ref: 17/LO/1565) and Cwm Taf University Health Board (17th November 2017, Ref: CT/831/205928/17).

Data sharing statement

No additional data are available.

For peer review only - http://bmjopen.bmj.com/site/about/guidelines.xhtml

References

1. World Health Organization. Primary Health Care - Now More Than Ever. Geneva: World Health Organization, 2008.
2. NHS England. General Practice Forward View. NHS England, 2016. https://www.england.nhs.uk/gp/gpfv/.
3. Mann C, Anderson C, Avery AJ, et al. Clinical pharmacists in general practice: pilot scheme. University of Nottingham, UK: NHS England, 2018. https://www.nottingham.ac.uk/pharmacy/documents/generalpracticeyearfwdrev/clinical-pharmacists-in-general-practice-pilot-scheme-full-report.pdf.
4. NHS England. Clinical pharmacists in general practice pilot. England, UK: NHS England, 2015. https://www.england.nhs.uk/gp/gpfv/workforce/building-the-general-practice-workforce/cp-gp/.
5. Barnes CB, Ulrik CS. Asthma and adherence to inhaled corticosteroids: current status and future perspectives. *Respir Care* 2015;60(3):455-68. doi: 10.4187/respcare.03200 [published Online First: 2014/08/15]
6. Murphy AC, Proeschal A, Brightling CE, et al. The relationship between clinical outcomes and medication adherence in difficult-to-control asthma. *Thorax* 2012;67(8):751-3. doi: 10.1136/thoraxjnl-2011-201096 [published Online First: 2012/03/23]
7. Cumella A. Falling through the gaps: Why more people need basic asthma care. United Kingdom: Asthma UK, 2017. https://www.asthma.org.uk/about/media/news/press-release-around-800000-brits-with-asthma-getting-poor-care/.
8. Mes MA, Katzer CB, Chan AHY, et al. Pharmacists and medication adherence in asthma: a systematic review and meta-analysis. *Eur*

For peer review only

For peer review only - http://bmjopen.bmj.com/site/about/guidelines.xhtml

Respir J 2018;52(2) doi: 10.1183/13993003.00485-2018 [published Online First: 2018/07/07]

9. Elliott R, Boyd MJ, Salema NE, et al. Supporting adherence for people starting a new medication for a long-term condition through community pharmacies: A pragmatic randomised controlled trial of the New Medicine Service. *BMJ quality & safety* 2016;25(10):747-58.

10. Latif A, Pollock K, Boardman HF. The contribution of the Medicines Use Review (MUR) consultation to counseling practice in community pharmacies. *Patient Educ Couns* 2011;83(3):336-44. doi: http://dx.doi.org/10.1016/j.pec.2011.05.007

11. Gann B. Understanding and using health experiences: the policy landscape. In: Ziebland S, Coulter A, Calabrese JD, et al., eds. Understanding and using health experiences: improving patient care. Oxford, United Kingdom: Oxford University Press 2013.

12. O'Brien BC, Harris IB, Beckman TJ, et al. Standards for reporting qualitative research: a synthesis of recommendations. *Acad Med* 2014;89(9):1245-51. doi: 10.1097/acm.0000000000000388 [published Online First: 2014/07/01]

13. Gidman W, Ward P, McGregor L. Understanding public trust in services provided by community pharmacists relative to those provided by general practitioners: a qualitative study. *BMJ Open* 2012;2(3)

14. Hall MA, Dugan E, Zheng B, et al. Trust in physicians and medical institutions: what is it, can it be measured, and does it matter? *Milbank Q* 2001;79(4):613-39. [published Online First: 2002/01/16]

15. Latif A, Boardman HF, Pollock K. Understanding the patient perspective of the English community pharmacy Medicines Use Review (MUR). *Res Social Adm Pharm* 2013;9(6):949-57. doi: http://dx.doi.org/10.1016/j.sapharm.2013.01.005

For peer review only - http://bmjopen.bmj.com/site/about/guidelines.xhtml

16. Tinelli M, Ryan M, Bond C. Patients' preferences for an increased pharmacist role in the management of drug therapy. *Int J Pharm Pract* 2009;17(5):275-82.
17. Latif A, Waring J, Watmough D, et al. Examination of England's New Medicine Service (NMS) of complex health care interventions in community pharmacy. *Res Social Adm Pharm* 2016;12(6):966-89. doi: http://dx.doi.org/10.1016/j.sapharm.2015.12.007
18. Bereznicki B, Peterson G, Jackson S, et al. Perceived feasibility of a community pharmacy-based asthma intervention: a qualitative follow-up study. *J Clin Pharm Ther* 2011;36(3):348-55.
19. Bradley F, Wagner AC, Elvey R, et al. Determinants of the uptake of medicines use reviews (MURs) by community pharmacies in England: a multi-method study. *Health Policy* 2008;88(2):258-68.
20. Naik Panvelkar P, Armour C, Saini B. Community pharmacy-based asthma services-what do patients prefer? *J Asthma* 2010;47(10):1085-93. doi: https://dx.doi.org/10.3109/02770903.2010.514638
21. Braun V, Clarke V. Using thematic analysis in psychology. *Qualitative Research in Psychology* 2006;3(2):77-101. doi: 10.1191/1478088706qp063oa
22. MacPhail C, Khoza N, Abler L, et al. Process guidelines for establishing Intercoder Reliability in qualitative studies. 2016;16(2):198-212. doi: 10.1177/1468794115577012
23. Sturges JE, Hanrahan KJ. Comparing Telephone and Face-to-Face Qualitative Interviewing: a Research Note. *Qualitative Research* 2004;4(1):107-18. doi: 10.1177/1468794104041110
24. Perepelkin J. Public Opinion of Pharmacists and Pharmacist Prescribing. 2011;144(2):86-93. doi: 10.3821/1913-701x-144.2.86
25. Boyd MJ, Elliott R, Barber N, et al. The impact of the New Medicines Service (NMS) in England on patients adherence to their medicines. *Int J Pharm Pract* 2014;22:66. doi: 10.1111/ijpp.12146

For peer review only - http://bmjopen.bmj.com/site/about/guidelines.xhtml

26. British Thoracic Society/Scottish Intercollegiate Guidelines Network. British guideline on the management of asthma: a national clinical guideline. Edinburgh: British Thoracic Society/Scottish Intercollegiate Guidelines Network, 2016. https://www.brit-thoracic.org.uk/quality-improvement/guidelines/asthma.

27. Centre for Pharmacy Postgraduate Education. Clinical pharmacists in general practice education. Manchester, United Kingdom: University of Manchester, 2018. https://www.cppe.ac.uk/career/cpgpe/clinical-pharmacists-in-general-practice-education.

28. Horne R, Weinman J. Self-regulation and self-management in asthma: exploring the role of illness perceptions and treatment beliefs in explaining non-adherence to preventer medication. *Psychol Health* 2002;17(1):17-32.

For peer review only - http://bmjopen.bmj.com/site/about/guidelines.xhtml

APPENDIX A: DESCRIPTION OF A PHARMACIST-LED ADHERENCE CONSULTATION DELIVERED IN GENERAL PRACTICE

We will describe this service as if you were receiving it.

The service

The service is delivered by a clinical pharmacist based at your local GP practice. The aim is to **help people with asthma get on better with their inhalers**. This means answering all of your questions and discussing all of your concerns about your inhalers in detail.

Pre-consultation

You are booked in for a consultation with the clinical pharmacist by the GP receptionist (just like you would with a GP appointment).

The main consultation

You have a one-to-one consultation with the pharmacist in a consultation room. They will begin with a **standard asthma review**. You will talk about your asthma control, recent symptoms, smoking history, and your asthma action plan. They will check your inhaler technique and lung function.

You will then have an **in-depth discussion about your inhalers**. You can ask any questions or mention any concerns you have about your inhalers. This could be anything from concerns about side effects or questions about how the medication works. The pharmacist will give you useful information and feedback that is **specifically suited to you**. They may use a short video or print-outs to guide their discussion with you.

At the end of the consultation, you will set an asthma-related goal with the pharmacist. This could be something like "less night time asthma symptoms". The pharmacist will advise you on how to achieve your personal goal using your inhalers.

The follow-up consultation

1 month later, you will see the pharmacist again for a short consultation (no more than 10 minutes). The pharmacist will briefly ask how your asthma has been and check your inhaler technique/lung function. You will then discuss how you are getting on with your medicines and your personal asthma goal. Based on this discussion, the pharmacist will give further recommendations regarding your medication.

For peer review only - http://bmjopen.bmj.com/site/about/guidelines.xhtml

APPENDIX B: INTERVIEW TOPIC GUIDE

Background and Previous Experience

1. To start the interview, could you tell me when you were diagnosed with asthma?

2. What type of medications are you currently prescribed for your asthma?

    a. How has your experience been with these medications? (Prompting to see if there are concerns, side effects etc.)

3. A lot of people with asthma have questions or concerns about their inhalers or tablets. What do you do when you have any questions or concerns specifically about your asthma medication?

    a. Prompts: do you do research online? Do you talk to friends and family? Do you go talk to a healthcare professional?
    b. Could you tell me a bit about why you prefer (insert route, e.g. researching online, talking to the GP, talking to family etc.) when it comes to your medication?

4. Have you ever talked to a pharmacist about your asthma medication?

    a. If **YES**: Could you walk me through what happened during that interaction?
        i. Where did it take place? (community pharmacist, hospital pharmacist etc.)
        ii. Prompts: what type of information did they give you? Were they able to answer your questions? Did you feel comfortable talking to them about your asthma medication? Did you feel like it was helpful for you?
        iii. Would you consider the pharmacist as a regular part of your asthma care team? Why or why not?

    b. If **NO**: Could you imagine a pharmacist as part of your regular asthma care team?

5. I'd now like to talk specifically about your preventer inhaler (brown inhaler, steroid inhaler). They're normally prescribed for daily or twice daily use. A lot of asthma patients we've talked to often don't take the inhaler as regularly as prescribed, is this something you've experienced as well?
    a. Could you tell me a little bit about why?

Pharmacist in general practice

Great, that's the first part of our interview. Now we're going to continue on to the part where I'd like to get your thoughts on pharmacist-led consultations in general practice. I've e-mailed you a description of the service already, but would you like to review it to refresh your memory?

For peer review only - http://bmjopen.bmj.com/site/about/guidelines.xhtml

Do you have any questions or anything you would like me to clarify before we move on to the questions?

6. What are your initial thoughts on this pharmacist medication support service?
    a. Explore which aspects of the service are most salient to the participant, use prompts below when needed.

- In your opinion, would this type of service be a useful addition to the care you get from your GP/nurse/asthma specialist?
    - Is this a type of service you could see yourself using? Why or why not?
    - Do you think this service is any different from the asthma care you already receive?
- Do you think this type of service would help you take your preventer inhaler more regularly?
- How do you feel about a pharmacist taking on this type of clinical role?
    - Do you think pharmacists have enough training for this type of work?
        - What type of knowledge would you expect the pharmacist to have?
    - How do pharmacists compare to your other healthcare professionals (e.g. GP or nurse)?
    - Do you think this service is any different from seeing a community pharmacist? (Are they familiar with the NMS and MUR?)
    - Would you feel comfortable talking to a pharmacist about your asthma and your medication?
    - Do you feel like you can trust a pharmacist in this type of role?
- How do you feel about having a pharmacist based in a GP surgery?
    - How would you feel about this pharmacist having access to your medical records?
- What do you think about seeing a pharmacist on an appointment basis?
- When you think about (concerns/side effects discussed for question 2a), do you think those types of questions or concerns could be addressed by a pharmacist?
    - What would be the ideal way to support you with those questions or concerns?
- Do you think this type of service is convenient for people with asthma?

For peer review only - http://bmjopen.bmj.com/site/about/guidelines.xhtml

Reporting checklist for qualitative study.

Based on the SRQR guidelines.

Instructions to authors

Complete this checklist by entering the page numbers from your manuscript where readers will find each of the items listed below.

Your article may not currently address all the items on the checklist. Please modify your text to include the missing information. If you are certain that an item does not apply, please write "n/a" and provide a short explanation.

Upload your completed checklist as an extra file when you submit to a journal.

In your methods section, say that you used the SRQRreporting guidelines, and cite them as:

O'Brien BC, Harris IB, Beckman TJ, Reed DA, Cook DA. Standards for reporting qualitative research: a synthesis of recommendations. Acad Med. 2014;89(9):1245-1251.

		Reporting Item	Page Number
**Title**			
	#1	Concise description of the nature and topic of the study identifying the study as qualitative or indicating the approach (e.g. ethnography, grounded theory) or data collection methods (e.g. interview, focus group) is recommended	0
**Abstract**			
	#2	Summary of the key elements of the study using the abstract format of the intended publication; typically includes background, purpose, methods, results and conclusions	1
**Introduction**			
Problem formulation	#3	Description and signifcance of the problem / phenomenon studied: review of relevant theory and empirical work; problem statement	2-3

For peer review only

Purpose or research question	#4	Purpose of the study and specific objectives or questions	3
**Methods**			
Qualitative approach and research paradigm	#5	Qualitative approach (e.g. ethnography, grounded theory, case study, phenomenolgy, narrative research) and guiding theory if appropriate; identifying the research paradigm (e.g. postpositivist, constructivist / interpretivist) is also recommended; rationale. The rationale should briefly discuss the justification for choosing that theory, approach, method or technique rather than other options available; the assumptions and limitations implicit in those choices and how those choices influence study conclusions and transferability. As appropriate the rationale for several items might be discussed together.	3
Researcher characteristics and reflexivity	#6	Researchers' characteristics that may influence the research, including personal attributes, qualifications / experience, relationship with participants, assumptions and / or presuppositions; potential or actual interaction between researchers' characteristics and the research questions, approach, methods, results and / or transferability	5
Context	#7	Setting / site and salient contextual factors; rationale	5
Sampling strategy	#8	How and why research participants, documents, or events were selected; criteria for deciding when no further sampling was necessary (e.g. sampling saturation); rationale	3-5
Ethical issues pertaining to human subjects	#9	Documentation of approval by an appropriate ethics review board and participant consent, or explanation for lack thereof; other confidentiality and data security issues	17
Data collection methods	#10	Types of data collected; details of data collection procedures including (as appropriate) start and stop dates of data collection and analysis, iterative process, triangulation of sources / methods, and modification of	5

For peer review only

		procedures in response to evolving study findings; rationale	
Data collection instruments and technologies	#11	Description of instruments (e.g. interview guides, questionnaires) and devices (e.g. audio recorders) used for data collection; if / how the instruments(s) changed over the course of the study	5
Units of study	#12	Number and relevant characteristics of participants, documents, or events included in the study; level of participation (could be reported in results)	6
Data processing	#13	Methods for processing data prior to and during analysis, including transcription, data entry, data management and security, verification of data integrity, data coding, and anonymisation / deidentification of excerpts	5
Data analysis	#14	Process by which inferences, themes, etc. were identified and developed, including the researchers involved in data analysis; usually references a specific paradigm or approach; rationale	5-6
Techniques to enhance trustworthiness	#15	Techniques to enhance trustworthiness and credibility of data analysis (e.g. member checking, audit trail, triangulation); rationale	6
**Results/findings**			
Syntheses and interpretation	#16	Main findings (e.g. interpretations, inferences, and themes); might include development of a theory or model, or integration with prior research or theory	7-13
Links to empirical data	#17	Evidence (e.g. quotes, field notes, text excerpts, photographs) to substantiate analytic findings	7-13
**Discussion**			
Intergration with prior work, implications, transferability and contribution(s) to the field	#18	Short summary of main findings; explanation of how findings and conclusions connect to, support, elaborate on, or challenge conclusions of earlier scholarship; discussion of scope of application / generalizability; identification of unique contributions(s) to scholarship in a discipline or field	13-16

For peer review only

Limitations	#19	Trustworthiness and limitations of findings	13-14
**Other**			
Conflicts of interest	#20	Potential sources of influence of perceived influence on study conduct and conclusions; how these were managed	17
Funding	#21	Sources of funding and other support; role of funders in data collection, interpretation and reporting	17

The SRQR checklist is distributed with permission of Wolters Kluwer © 2014 by the Association of American Medical Colleges. This checklist was completed on 01. June 2019 using https://www.goodreports.org/, a tool made by the EQUATOR Network in collaboration with Penelope.ai

For peer review only

BMJ Open

Pharmacist-led adherence support in general practice: a qualitative interview study of adults with asthma

Journal:	*BMJ Open*
Manuscript ID	bmjopen-2019-032084.R2
Article Type:	Original research
Date Submitted by the Author:	22-Sep-2019
Complete List of Authors:	Mes, Marissa; UCL School of Pharmacy, Centre for Behavioural Medicine, Practice & Policy<br>Katzer, Caroline; UCL School of Pharmacy, Centre for Behavioural Medicine, Practice & Policy<br>Wileman, Vari; UCL School of Pharmacy, Centre for Behavioural Medicine, Practice & Policy<br>Chan, Amy; UCL School of Pharmacy, Centre for Behavioural Medicine, Practice & Policy<br>Horne, Robert; UCL School of Pharmacy, Centre for Behavioural Medicine, Practice & Policy<br>Taylor, Stephanie; Queen Mary University of London, Centre for Primary Care and Public Health
**Primary Subject Heading**:	General practice / Family practice
Secondary Subject Heading:	Respiratory medicine, Health services research
Keywords:	Medication Adherence, Asthma < THORACIC MEDICINE, Pharmaceutical Services, General Practice, Medication Therapy Management

SCHOLARONE™
Manuscripts

Pharmacist-led adherence support in general practice: a qualitative interview study of adults with asthma

Mes, Marissa Ayano[1]; Katzer, Caroline Brigitte[1]; Wileman, Vari[1]; Chan, Amy Hai Yan[1]; Horne, Rob[1]; & Taylor, Stephanie Jane Caroline[2].

[1]Centre for Behavioural Medicine, UCL School of Pharmacy, Mezzanine Floor, Tavistock Square, London WC1H 9JP, London, United Kingdom.

[2]Centre for Primary Care and Public Health, Blizard Institute, Queen Mary University London, Yvonne Carter Building, 58 Turner Street, London E1 2AB, United Kingdom.

Corresponding author: Marissa Ayano Mes (marissa.mes.15@ucl.ac.uk)

Word count: 3,944 words

For peer review only

Abstract

Objectives: The National Health Service (NHS) in England recently introduced general practice pharmacists (GPPs) to provide medication-focused support to both patients and the general practice team. This healthcare model may benefit people with asthma, who currently receive sub-optimal care and demonstrate low medication adherence. This study aimed to explore the perspectives of adults with asthma on the potential for pharmacist-led adherence support delivered in general practice, with a focus on how these perspectives are formed.

Design and setting: The study was conducted in the United Kingdom (UK) utilising a qualitative interview methodology. Participants were invited to partake in a telephone-based semi-structured interview, followed by an online questionnaire for demographic details and asthma history. Qualitative data was analysed using thematic analysis.

Participants: Participants (n = 17) were adults with asthma in the UK with a prescription for an inhaled corticosteroid. Participants did not have previous experience with GPPs and were asked to provide their views on a proposed GPP-led service.

Results: Participant perspectives of GPPs were determined by trust in pharmacists, perceived gaps in asthma care, and the perceived strain on the NHS. Trust was based on pharmacists' perceived clinical competency, established over time, and gauged through a 'benchmarking' process. GPP's fit in current asthma care was assessed based on potential role overlap with other healthcare professionals, continuity of care, and medication-related support needs. Participants navigated the NHS based on a perceived hierarchy of healthcare professionals (GPs on top, nurses, then pharmacists), and this influenced their perspectives of GPPs.

Conclusions: While the GPP scheme shows promise based on the perspectives of people with asthma, the identified barriers to optimal patient engagement and service implementation will need to be addressed for the service to be effective.

For peer review only - http://bmjopen.bmj.com/site/about/guidelines.xhtml

Strengths and limitations of this study

- The use of qualitative methodology captured the complex processes behind people's perspectives of general practice pharmacists (GPPs), including how lived experiences shaped opinions of new pharmacist-led services.
- Telephone-based interviews enabled recruitment across the United Kingdom (UK) and increased study accessibility for people with severe asthma and travel limitations.
- The study may not have captured the full variation in views among adults with asthma in the UK because participants primarily had self-reported mild asthma and were recruited through an asthma charity.
- Participants had no experience with GPP-led consultations and therefore represented the general population with asthma that would initially need to be convinced to engage with the service.

Background

The pressure on primary care to deliver core services is increasing rapidly worldwide due to a growing and ageing population, the prevalence of long-term conditions, and significant resource constraints. Primary care systems are being reshaped and new models of care are emerging to cope with growing demand.[1]

One such model is the general practice pharmacist (GPP) model, which was introduced in England as part of the NHS *General Practice Forward View* initiative.[2-4] These pharmacists support both patients and the general practice team with medication-related issues, with the aim of expanding the general practice workforce, reducing practice burden, and increasing patient access to appointments.[4] Initial qualitative feedback from general practitioners (GPs) and pharmacists in a pilot study in England suggests that GPPs can have positive impacts on

For peer review only - http://bmjopen.bmj.com/site/about/guidelines.xhtml

medication safety, medication adherence, healthcare access, and patient satisfaction across a variety of long-term conditions.[3]

People with asthma may benefit from GPP support. Research shows that adherence to inhaled corticosteroids (ICS), an essential medication for asthma, is low.[5 6] Furthermore, only 35% of people with asthma in the United Kingdom (UK) receive basic care (i.e. annual reviews, inhaler technique checks, and a written asthma action plan).[7] Previous research suggests that pharmacist-led interventions can increase medication adherence and support asthma self-management among people with asthma.[8] However, most of the previous research in the UK has been limited to community rather than general practice pharmacists, and focused on their impact across a range of long-term conditions rather than asthma specifically.[9 10]

Understanding the specific perspectives of people with asthma regarding GPPs is an important first step in establishing the potential benefits of this new service for this specific patient group, as well as identifying any potential issues in its future uptake and effectiveness.[11] The aim of this study was to explore the perspectives of adults with asthma on the potential of pharmacist-led asthma adherence support delivered in general practice, with a focus on how these perspectives are formed.

Methods

This study is reported according to the Standards for Reporting Qualitative Research.[12] It was a telephone-based semi-structured interview study, using an interpretivist approach to understand how adults with asthma construct their initial opinions of GPP-led asthma adherence support. Demographic details and asthma history were collected using a brief online questionnaire.

For peer review only - http://bmjopen.bmj.com/site/about/guidelines.xhtml

Participants

Participants were adults (≥ 18 years old) living in the UK and proficient in English, with a self-reported asthma diagnosis, a prescription for ICS, and access to a telephone and e-mail account. People with respiratory comorbidities (e.g. Chronic Obstructive Pulmonary Disease) and/or those in hospital or nursing homes were excluded, as the adherence behaviour and support needs of these individuals were hypothesised to be different.

Recruitment

Several recruitment channels were used to ensure that participants varied in age, gender, and self-reported asthma severity. A flyer with study information and researchers' contact details was circulated by researchers, the Asthma UK Centre for Applied Research (AUKCAR), and the National Institute for Health Research Collaboration for Leadership in Applied Health Research and Care North Thames via social media. The study was advertised in two electronic newsletters: the Asthma UK volunteer bulletin and the University College London student newsletter. Printed flyers were handed directly to potential participants by a respiratory consultant at a London hospital, and pharmacists at two hospitals in Wales.

People who contacted the researchers were e-mailed an information sheet, consent form, and eligibility criteria to review. If they were eligible and willing to participate, they were booked in for a one-hour telephone interview. In preparation for the call, participants were asked to read a description of a GPP-led adherence support consultation, sent to them via e-mail (see Appendix A). This description was based on the work of a clinical respiratory pharmacist working in general practice in London. Participants gave verbal consent over the telephone before the interview began. The consent procedure and interview were audio-recorded with their permission. All participants received a £20 online shopping voucher to thank them for their time.

For peer review only

Data collection and analysis

One researcher (MM) conducted the interviews. Participants were informed that the researcher had a background in Health Psychology and an interest in adherence and pharmacist-led care for asthma.

The interview topic guide had two sections (see Appendix B). The first section focused on participants' previous experiences of asthma, asthma care, and pharmacists. The second section focused on how these lived experiences informed participants' opinions of GPP-led adherence support, with questions based on previous research on interpersonal/institutional trust in healthcare professionals,[13 14] perceptions of the pharmacist role,[15-17] and pharmacist-led care for asthma.[10 18-20]

Participants also completed an online questionnaire on demographic details (gender and age) and asthma history (self-reported asthma severity, hospitalisations, and GP visits). An online questionnaire was used because participants may have felt uncomfortable disclosing personal information (e.g. age) directly to a researcher during the telephone call. The self-report method was chosen because recruitment to the study may have been difficult if access to participants' medical records was required.

All interviews were professionally transcribed, with transcripts checked for accuracy by MM. Data were analysed using NVivo (QSR, Version 11). Thematic analysis was used to identify themes at the semantic level using a deductive approach, based on their relevance to the study aim.[21] Continuous iterative analyses were conducted to establish when thematic saturation had been reached. Recruitment was set for 30 participants or thematic saturation, whichever was attained first. The study ran from October 2017 to February 2018. All transcripts were analysed by MM, and four transcripts (representing 25% of the total transcripts) were

For peer review only - http://bmjopen.bmj.com/site/about/guidelines.xhtml

independently second-coded by another researcher (CK), as recommended by MacPhail, et al. [22]. Discrepancies were resolved through consensus discussion.

Patient and public involvement

All study materials (recruitment flyer, participant information sheet, consent form, interview topic guide, GPP consultation example, and online questionnaire) were reviewed by the AUKCAR Patient Advisory Group (PAG) prior to study commencement. Members of the PAG were adults with asthma. Their feedback, often regarding word choice and text length, was incorporated into the study materials.

Results

Thematic saturation was reached with 17 participants (Table 1). The median interview length was 42 minutes (ranging from 30 to 58 minutes). The participant sample was 59% female, with most participants (41%) aged 30 to 39 years and recruited through the Asthma UK newsletter (41%). The sample included participants with self-reported mild (53%), moderate (24%), and severe asthma (17%). Three overarching themes (with seven sub-themes) were identified from the data: building trust in pharmacists, filling gaps in current asthma care, and navigating a strained healthcare system.

Table 1. Demographic characteristics and asthma history

Characteristics (n = 17)	Frequency n (%)
**Gender**	
Female	10 (59%)
Male	7 (41%)
**Age in years**	
18 – 29	5 (29%)
30 – 39	7 (41%)
40 – 49	2 (12%)
50 - 59	-
60 – 69	2 (12%)
70 +	1 (6%)
**Recruitment channel**	
Asthma UK newsletter	7 (41%)

For peer review only - http://bmjopen.bmj.com/site/about/guidelines.xhtml

Hospital	1 (6%)
Social media	3 (17%)
University College London newsletter	3 (18%)
Word of Mouth	3 (18%)
**Self-reported asthma severity**	
Mild	9 (53%)
Moderate	4 (24%)
Severe	3 (17%)
Prefer not to disclose	1 (6%)
**Self-reported hospitalisations for asthma (previous 12 months)**	
0	11 (65%)
1 – 4	3 (17%)
5 – 10	3 (17%)
**Self-reported GP visits for asthma (previous 12 months)**	
0	2 (12%)
1 – 10	13 (76%)
10 – 20	2 (12%)

Theme 1: building trust in pharmacists

Trust in healthcare professionals involves the optimistic acceptance of being in a vulnerable situation, knowing that one's interests will be cared for.[14] For participants, opinions of the new service were based on the level of trust they placed in pharmacists. Trust was built over time, based on perceived clinical competency, and through a benchmarking process, which form the sub-themes discussed below.

Building trust over time

Participants highlighted that trust in any healthcare professional builds through consistent contact over time. Some participants felt hesitant about the new service because it meant deviating from their usual trusted healthcare professional, suggesting a preference for usual care over new initiatives to maintain the quality of their asthma care.

"I don't really know, I think I'd prefer a doctor [to talk to about my asthma]. It's the way it's always been."

– P15, male, 30 – 39 years, mild asthma

For peer review only - http://bmjopen.bmj.com/site/about/guidelines.xhtml

Building trust based on perceived clinical competency

When asked about specific criteria for trust, participants discussed pharmacists' clinical competency. This included pharmacists' asthma-specific and broad clinical knowledge. Support for the new service was high when pharmacists were viewed as knowledgeable.

"So I know that in the pharmacy role they're very knowledgeable. So if [adherence support for asthma] is something they want to do then why not? I have a lot of faith in somebody who's got a lot of knowledge in something."
– P8, female, 30 – 39 years, severe asthma

Some participants believed that pharmacists would need extensive additional training to develop the knowledge needed for the new service. Their main concern was that pharmacists were too medication-focused and therefore lacked broader clinical skills.

"…it could be that the [medication] side-effects are something else entirely. So [pharmacists] would be kind of completely thinking down the asthma route, 'it might just be that you're taking an inhaler that you feel side-effects'…but what if it turns out you actually have cancer?"
– P17, male, 18 – 29 years, mild asthma

Building trust through a benchmarking process

None of the participants had previous experiences with GPPs, with some participants questioning the differences between GPPs and community pharmacists. Many participants engaged in a *benchmarking* process: using their trust and previous experiences of other healthcare professionals to gauge how much they could trust a GPP. Common reference points included community pharmacists, respiratory consultants, nurses, GPs, and paramedics.

"…I know [pharmacist support] is there but I still don't understand it with [brittle] asthma because I still get wary. If paramedics have never heard of it and don't know what they're doing, how's a pharmacist going to hear of it?"
– P2, female, 30 – 39 years, severe asthma

"I would much rather go to a pharmacist than to a nurse to discuss the medication issues that I was having… I can see an asthma nurse to discuss medication, and I was like 'Really? What

For peer review only - http://bmjopen.bmj.com/site/about/guidelines.xhtml

do nurses know about…not to be rude, but what do they know about medication more than my specialist who prescribed it?"

– P4, female, 30 – 39 years, mild asthma

Theme 2: filling gaps in current asthma care

Participants' opinions of GPPs were also informed by perceived gaps in their current asthma care. Participants evaluated the new service's place in their current care based on potential role overlap between GPPs and other healthcare professionals, continuity of care, and medication-specific support.

Potential role overlap

Participants that saw potential role overlap between GPPs and other healthcare professionals were more sceptical of the new service.

"I think if I was having an annual asthma review I wouldn't need to use the pharmacist's service as well, but it might be an alternative to the annual asthma review…"

– P3, male, 70+ years, mild asthma

However, other participants clearly delineated the GPP role, and these participants often recommended ways to integrate pharmacists into their care.

"If you're asking a GP, you've got maybe five, ten minutes… If you know you've got another ten or fifteen minutes with this pharmacist… for asking all these questions…with the GP you can concentrate on the problem and get that sorted, and then go see the pharmacist and discuss the medication."

– P5, female, 60 – 69 years, moderate asthma

Continuity of care

Participants with self-reported severe asthma often had multiple healthcare professionals involved in their care (e.g. respiratory consultants, GPs, and asthma nurses). When asked about the new service, some participants felt concerned about involving an additional healthcare professional in their care. This was unrelated to their views on pharmacist

For peer review only - http://bmjopen.bmj.com/site/about/guidelines.xhtml

competency, and was usually influenced by previous experiences of inadequate continuity of care due to a lack of communication between healthcare professionals.

"[Pharmacists] always say speak to your GP but then the GP tells you to speak to the pharmacist because they're supposed to know more about drugs than what they are…and then you're somewhere in the middle…"
– P2, female, 30 – 39 years, severe asthma

Medication-specific support

Other participants with severe asthma who spoke about being on multiple medications and/or having other health concerns welcomed the service. This enthusiasm came from the fact that they identified gaps in their current care that they believed could be filled by pharmacists as medication experts.

"…just having contact with someone who actually…knows about the medication, like they know how they work and what the potential side effects are going to be and interactions…it's that knowledge that a GP wouldn't necessarily have time to tell you all about…"
– P6, female, 30 – 39 years, severe asthma

"…I'm trying to conceive at the moment so…and I thought I don't want to be taking anything that's unnatural or steroid-y…I did ask the respiratory consultant [about asthma medications and In Vitro Fertilisation] but he didn't know…"
–P8, female, 30 – 39 years, severe asthma

Some participants felt that GPPs should have an independent prescribing qualification to fulfil their role as medication experts. They worried that the new service might contribute to the burden on patients and/or the healthcare system, and that independent pharmacist prescribers would minimise the risk of this happening.

"For me, it would just be down to whether or not [pharmacists] are able to prescribe. I don't imagine that they wouldn't have the knowledge that was required…It's just if I had to then see a doctor to be prescribed a different medication, I'd rather just go to see the doctor instead."
– P10, female, 40 – 49 years, mild asthma

For peer review only - http://bmjopen.bmj.com/site/about/guidelines.xhtml

Theme 3: navigating a strained healthcare system

Participants were acutely aware of the limited resources within general practice. They often expressed guilt and frustration about booking appointments for asthma. Participants never booked appointments just for medication-related questions, and their medication-related concerns were frequently left unaddressed because other topics took priority in a consultation, particularly if the participant had multiple comorbidities. The pharmacist-led service was welcomed by these participants because they felt pharmacists would have more time to focus on their medication.

"…come Monday morning I wouldn't want to call the GP because I know on Monday morning they're very, very busy…I'll just sort of crack on at home, multi-dosing salbutamol and seeing what happens."
– P8, female, 30 – 39 years, severe asthma

"[GPs] just want you in and out… 'oh yes, I wanted to ask you something else' but too late now, you're away. That's how you feel."
–P1, female, 30 – 39 years, mild asthma

The hierarchy of healthcare professionals

Many participants constructed a hierarchy of healthcare professionals with GPs at the top, followed by nurses and finally pharmacists. This hierarchy determined the importance of each healthcare professional's time. Severe health concerns justified booking a GP appointment, while non-urgent concerns were viewed as more suitable for pharmacists.

The hierarchy of healthcare professionals affected opinions of GPPs in both directions. Some participants were enthusiastic about the new service because they believed it would lessen the workload of GPs and nurses. For these participants, seeing a pharmacist (the healthcare professional further down the hierarchy) felt less intimidating and formal, slightly easing concerns about taking up valuable appointment time.

For peer review only - http://bmjopen.bmj.com/site/about/guidelines.xhtml

For peer review only

"…to be honest, GPs have bigger problems to deal with…[they're] dealing with people with, you know, life threatening illnesses, then actually seeing the standard case of asthma or an asthma check-up isn't the best use of [their] time."
– P16, male, 18 – 29 years, moderate asthma

"It feels less formal, I think, when you're with a pharmacist than when you're in the doctor's…sometimes when you go to the doctor's, you're kind of clock watching…"
– P10, female, 40 – 49 years, mild asthma

Others felt that pharmacists could not extend into a clinical role similar to GPs and nurses, with some suggesting a triage-like function to safeguard GP time.

"I never feel as though a pharmacist is a nurse, if you see what I mean. A nurse has practical hands-on experience of trying to make people better. The pharmacist is one who deals with the theory of medication."
–P9, male, 60 – 69 years, undisclosed asthma severity

"… the pharmacist has seen you and if there's communication between the pharmacist and the GP, so that I guess it would help the GPs prioritise who they saw…"
–P11, female, 40 – 49 years, moderate asthma

However, pharmacists themselves were also viewed as a limited resource. Many participants supported moving pharmacists from community pharmacies to general practice because they experienced inadequate care in busy community pharmacies. Others were concerned that pharmacist-led adherence support with a wide scope (i.e. for multiple long-term conditions) would limit access for people with asthma.

"If [pharmacists] weren't running a community pharmacy, if they were linked in, if they worked within the GP surgery with a lot of time, then yes, I don't see how [a lack of time] would be an issue."
– P12, female, 30 – 39 years, mild asthma

"…my worry is if a pharmacist has to do [adherence support in general practice] for asthma, what other long-term conditions will they have to do it for?"
– P6, female, 30 – 39 years, severe asthma

Discussion

This is the first in-depth exploration of the perspectives of adults with asthma on pharmacist-led adherence support in general practice. This focused exploration identified potential

For peer review only - http://bmjopen.bmj.com/site/about/guidelines.xhtml

barriers to service uptake, and has the potential to help further refine and tailor the GPP service as it is rolled out.

Interpretation of findings

Trust played an important role in participants' initial perspectives of GPP-led care – it was an essential component of the patient-pharmacist relationship and it informed participants' views of pharmacists' role in asthma care. These findings suggest that general awareness of the existence of pharmacist-led services is insufficient to encourage service uptake.

Participants based their trust in pharmacists on perceived clinical competency and comparisons with other trusted healthcare professionals (e.g. GPs and nurses), guided by perceptions of pharmacists' position in the hierarchy of healthcare professionals. These findings may suggest that explicit endorsement of GPP-led care by other trusted healthcare professionals might improve service uptake among adults with asthma. Reassurance and support from GPs and nurses may address some of the concerns raised by study participants, including those about fragmented care, pharmacists' clinical competency, and potential role overlap.

Support for GPP-led care did not seem to differ based on participants' age, gender, or self-reported asthma severity – there was a variety of views in each group of participants. However, participants who spoke about being on multiple medications and/or having additional health concerns seemed to be more open to pharmacist input, perhaps because they felt current asthma care was unable to meet these additional needs. Targeting this group of adults with asthma may improve service uptake in the future.

For peer review only - http://bmjopen.bmj.com/site/about/guidelines.xhtml

Strengths and limitations

The qualitative method in this study captured the complex processes behind people's initial opinions of GPPs. The combination of recruitment channels produced variation in the sample in terms of age, self-reported asthma severity, and healthcare utilisation. Telephone-based interviews enabled recruitment across the UK without increasing participant burden, thus increasing study accessibility for participants with severe asthma and limited travel capacity. In addition, telephone-based interviews can produce data of higher quality compared to face-to-face interviews when sensitive topics (e.g. long-term conditions) are discussed.[23]

The participant sample may however not have captured the views of all adults with asthma because it consisted primarily of people with self-reported mild asthma (53%) and people recruited through Asthma UK (41%). Thematic saturation may have been reached due to the relative homogeneity of the participant sample. The participants recruited through Asthma UK may have had a stronger interest in asthma care or pharmacist-led support, and may therefore have been more supportive of the new service compared to the general population with asthma. However, if scepticism of the new service exists among people who are more engaged in their care, then the findings may be amplified in the general population with asthma who may have less interest in asthma care.

A major drawback of the study is that none of the participants had experienced a GPP consultation directly, with some participants recruited from Scotland and Northern Ireland where the GPP scheme does not exist. While the consultation description that participants were asked to read was based on real work by a clinical respiratory pharmacist working in general practice, study findings can only be used to understand patients' initial views of a GPP-led service. These participants represent the general population with asthma who would initially need to be convinced to engage with the service. Addressing some of the concerns

For peer review only - http://bmjopen.bmj.com/site/about/guidelines.xhtml

identified by participants may help improve the uptake of existing GPP-led care among adults with asthma. However, participants' views may change over time, and could potentially be influenced by direct personal experience with a GPP consultation.

Comparison with existing literature

There is limited research on patient perspectives of GPPs because the care model is relatively new. However, findings from this study align with those from community pharmacy-based research, suggesting that people with asthma may not differentiate between pharmacy sectors.

Findings align with work by Gidman, et al. [13], who found that people were hesitant about deviating from their usual trusted care model (often a GP). However, two of the older participants in this study were open to GPP-led care, in contrast to previous research that suggests that older patients may be less likely to accept an expanded pharmacist role.[24]

In line with the present study's findings, previous research with general members of the public and people with asthma found that other trusted healthcare professionals (e.g. GPs and nurses) were used as benchmarks to inform opinions of community pharmacist-led services.[13 20] Similarly, Naik Panvelkar, et al. [20] found that previous positive experiences with community pharmacists raised expectations for other pharmacist-led services in a population of people with asthma.

Participants' views of the gaps in their current asthma care shaped their perspectives of GPPs. Similarly, Boyd, et al. [25] found that recipients of the New Medicine Service (NMS) welcomed pharmacists' recommendations if they addressed a concern directly raised by the patient. While asthma care guidelines recommend a multidisciplinary approach in treating difficult asthma, the present study suggests that some people with self-reported severe asthma were hesitant to include another healthcare professional due to issues with continuity of care.[26] In line with previous research, the hierarchy of healthcare professionals influenced

For peer review only - http://bmjopen.bmj.com/site/about/guidelines.xhtml

For peer review only

perspectives of pharmacists expanding further into clinical roles.[13 15] However, this study also found that the hierarchy increased support for pharmacist-led care to reduce the burden on GPs/nurses.

Implications for research and practice

As the GPP model is rolled out, future studies could be conducted using in-depth interviews with people with asthma after they have experienced a GPP-led consultation. These interviews could establish if interpersonal factors (i.e. rapport with the pharmacist) have an impact on patient perspectives. Ethnographic observations of pharmacist-led consultations and the general practice team will help assess pharmacists' integration and its effect on continuity of care for asthma patients. Future recruitment should aim for greater variation in participants (e.g. self-reported asthma severity) through various recruitment channels, with additional efforts to look for discordant voices when thematic saturation is reached.

Findings from this study could be implemented in efforts to increase service uptake among people with asthma. Given the *benchmarking* process used to establish trust in pharmacists, comparisons between GPPs and other healthcare professionals could be used to inform and engage the public. For example, public campaigns highlighting the differences and similarities between GPs and GPPs may help the public differentiate the pharmacist role and understand the added value of the new service within asthma care.

Participants wanted GPPs to have broad clinical skills and a prescribing qualification. Encouragingly, the Centre for Pharmacy Postgraduate Education has already included these components in their GPP training pathways.[27]

Although the hierarchy of healthcare professionals sometimes prevented pharmacists from being perceived as clinicians, it also made GPP appointments appear less formal and intimidating to access. Participants felt more comfortable making an appointment with a

For peer review only - http://bmjopen.bmj.com/site/about/guidelines.xhtml

pharmacist for medication-related questions. This is encouraging because the new service may encourage people with asthma to address medication-related concerns that may be barriers to medication adherence.[28]

While the perspectives of people with asthma explored in this study show that the GPP model has promise, they identified several barriers to optimal patient engagement and service implementation that will need to be addressed for the service to be effective. Meeting patient expectations will be the first crucial step in ensuring the programme's long-term benefit and reducing the pressure on general practice in England.

Acknowledgements

We would like to thank the Asthma UK Centre for Applied Research (AUKCAR) Patient Advisory Group for their feedback and support for this study.

Contributors

The study was designed by MM, RH, and SJCT. All interviews were conducted by MM. Data was analysed by MM and CK. The manuscript was written by MM, AHYC, and VW, with input from RH and SJCT.

Funding

The research was funded by the National Institute for Health Research (NIHR) Collaboration for Leadership in Applied Health Research and Care North Thames at Barts Health NHS Trust. The views expressed are those of the authors and not necessarily those of the NHS, the NIHR, or the Department of Health and Social Care.

Competing interests

None declared.

For peer review only - http://bmjopen.bmj.com/site/about/guidelines.xhtml

Patient consent for publication

Not required.

Ethics approval

The research was approved by the NHS London-Harrow Research Ethics Committee (12th October 2017, Ref: 17/LO/1565) and Cwm Taf University Health Board (17th November 2017, Ref: CT/831/205928/17).

Data sharing statement

No additional data are available.

For peer review only

For peer review only - http://bmjopen.bmj.com/site/about/guidelines.xhtml

References

1. World Health Organization. Primary Health Care - Now More Than Ever. Geneva: World Health Organization, 2008.
2. NHS England. General Practice Forward View. NHS England, 2016. https://www.england.nhs.uk/gp/gpfv/.
3. Mann C, Anderson C, Avery AJ, et al. Clinical pharmacists in general practice: pilot scheme. University of Nottingham, UK: NHS England, 2018. https://www.nottingham.ac.uk/pharmacy/documents/generalpracticeyearfwdrev/clinical-pharmacists-in-general-practice-pilot-scheme-full-report.pdf.
4. NHS England. Clinical pharmacists in general practice pilot. England, UK: NHS England, 2015. https://www.england.nhs.uk/gp/gpfv/workforce/building-the-general-practice-workforce/cp-gp/.
5. Barnes CB, Ulrik CS. Asthma and adherence to inhaled corticosteroids: current status and future perspectives. *Respir Care* 2015;60(3):455-68. doi: 10.4187/respcare.03200
6. Murphy AC, Proeschal A, Brightling CE, et al. The relationship between clinical outcomes and medication adherence in difficult-to-control asthma. *Thorax* 2012;67(8):751-3. doi: 10.1136/thoraxjnl-2011-201096
7. Cumella A. Falling through the gaps: Why more people need basic asthma care. United Kingdom: Asthma UK, 2017. https://www.asthma.org.uk/about/media/news/press-release-around-800000-brits-with-asthma-getting-poor-care/.
8. Mes MA, Katzer CB, Chan AHY, et al. Pharmacists and medication adherence in asthma: a systematic review and meta-analysis. *Eur Respir J* 2018;52(2) doi: 10.1183/13993003.00485-2018
9. Elliott R, Boyd MJ, Salema NE, et al. Supporting adherence for people starting a new medication for a long-term condition through community pharmacies: A pragmatic randomised controlled trial of the New Medicine Service. *BMJ quality & safety* 2016;25(10):747-58. doi: 10.1136/bmjqs-2015-004400
10. Latif A, Pollock K, Boardman HF. The contribution of the Medicines Use Review (MUR) consultation to counseling practice in community pharmacies. *Patient Educ Couns* 2011;83(3):336-44. doi: 10.1016/j.pec.2011.05.007
11. Gann B. Understanding and using health experiences: the policy landscape. In: Ziebland S, Coulter A, Calabrese JD, et al., eds. Understanding and using health experiences: improving patient care. Oxford, United Kingdom: Oxford University Press 2013.

For peer review only - http://bmjopen.bmj.com/site/about/guidelines.xhtml

12. O'Brien BC, Harris IB, Beckman TJ, et al. Standards for reporting qualitative research: a synthesis of recommendations. *Acad Med* 2014;89(9):1245-51. doi: 10.1097/acm.0000000000000388
13. Gidman W, Ward P, McGregor L. Understanding public trust in services provided by community pharmacists relative to those provided by general practitioners: a qualitative study. *BMJ Open* 2012;2(3) doi: 10.1136/bmjopen-2012-000939
14. Hall MA, Dugan E, Zheng B, et al. Trust in physicians and medical institutions: what is it, can it be measured, and does it matter? *Milbank Q* 2001;79(4):613-39. doi: 10.1111/1468-0009.00223
15. Latif A, Boardman HF, Pollock K. Understanding the patient perspective of the English community pharmacy Medicines Use Review (MUR). *Res Social Adm Pharm* 2013;9(6):949-57. doi: 10.1016/j.sapharm.2013.01.005
16. Tinelli M, Ryan M, Bond C. Patients' preferences for an increased pharmacist role in the management of drug therapy. *Int J Pharm Pract* 2009;17(5):275-82. doi: 10.1211/ijpp.17.05.0004
17. Latif A, Waring J, Watmough D, et al. Examination of England's New Medicine Service (NMS) of complex health care interventions in community pharmacy. *Res Social Adm Pharm* 2016;12(6):966-89. doi: 10.1016/j.sapharm.2015.12.007
18. Bereznicki B, Peterson G, Jackson S, et al. Perceived feasibility of a community pharmacy-based asthma intervention: a qualitative follow-up study. *J Clin Pharm Ther* 2011;36(3):348-55. doi: 10.1111/j.1365-2710.2010.01187.x
19. Bradley F, Wagner AC, Elvey R, et al. Determinants of the uptake of medicines use reviews (MURs) by community pharmacies in England: a multi-method study. *Health Policy* 2008;88(2):258-68. doi: 10.1016/j.healthpol.2008.03.013
20. Naik Panvelkar P, Armour C, Saini B. Community pharmacy-based asthma services-what do patients prefer? *J Asthma* 2010;47(10):1085-93. doi: 10.3109/02770903.2010.514638
21. Braun V, Clarke V. Using thematic analysis in psychology. *Qualitative Research in Psychology* 2006;3(2):77-101. doi: 10.1191/1478088706qp063oa
22. MacPhail C, Khoza N, Abler L, et al. Process guidelines for establishing Intercoder Reliability in qualitative studies. *Qualitative Research* 2016;16(2):198-212. doi: 10.1177/1468794115577012
23. Sturges JE, Hanrahan KJ. Comparing Telephone and Face-to-Face Qualitative Interviewing: a Research Note. *Qualitative Research* 2004;4(1):107-18. doi: 10.1177/1468794104041110
24. Perepelkin J. Public Opinion of Pharmacists and Pharmacist Prescribing. *Canadian Pharmacists Journal* 2011;144(2):86-93. doi: 10.3821/1913-701x-144.2.86

For peer review only - http://bmjopen.bmj.com/site/about/guidelines.xhtml

25. Boyd MJ, Elliott R, Barber N, et al. The impact of the New Medicines Service (NMS) in England on patients adherence to their medicines. *Int J Pharm Pract* 2014;22:66. doi: 10.1111/ijpp.12146
26. British Thoracic Society/Scottish Intercollegiate Guidelines Network. British guideline on the management of asthma: a national clinical guideline. Edinburgh: British Thoracic Society/Scottish Intercollegiate Guidelines Network, 2016. https://www.brit-thoracic.org.uk/quality-improvement/guidelines/asthma.
27. Centre for Pharmacy Postgraduate Education. Clinical pharmacists in general practice education. Manchester, United Kingdom: University of Manchester, 2018. https://www.cppe.ac.uk/career/cpgpe/clinical-pharmacists-in-general-practice-education.
28. Horne R, Weinman J. Self-regulation and self-management in asthma: exploring the role of illness perceptions and treatment beliefs in explaining non-adherence to preventer medication. *Psychol Health* 2002;17(1):17-32. doi: 10.1080/08870440290001502

For peer review only

For peer review only - http://bmjopen.bmj.com/site/about/guidelines.xhtml

APPENDIX A: DESCRIPTION OF A PHARMACIST-LED ADHERENCE CONSULTATION DELIVERED IN GENERAL PRACTICE

We will describe this service as if you were receiving it.

The service

The service is delivered by a clinical pharmacist based at your local GP practice. The aim is to **help people with asthma get on better with their inhalers**. This means answering all of your questions and discussing all of your concerns about your inhalers in detail.

Pre-consultation

You are booked in for a consultation with the clinical pharmacist by the GP receptionist (just like you would with a GP appointment).

The main consultation

You have a one-to-one consultation with the pharmacist in a consultation room. They will begin with a **standard asthma review**. You will talk about your asthma control, recent symptoms, smoking history, and your asthma action plan. They will check your inhaler technique and lung function.

You will then have an **in-depth discussion about your inhalers**. You can ask any questions or mention any concerns you have about your inhalers. This could be anything from concerns about side effects or questions about how the medication works. The pharmacist will give you useful information and feedback that is **specifically suited to you**. They may use a short video or print-outs to guide their discussion with you.

At the end of the consultation, you will set an asthma-related goal with the pharmacist. This could be something like “less night time asthma symptoms”. The pharmacist will advise you on how to achieve your personal goal using your inhalers.

The follow-up consultation

1 month later, you will see the pharmacist again for a short consultation (no more than 10 minutes). The pharmacist will briefly ask how your asthma has been and check your inhaler technique/lung function. You will then discuss how you are getting on with your medicines and your personal asthma goal. Based on this discussion, the pharmacist will give further recommendations regarding your medication.

For peer review only - http://bmjopen.bmj.com/site/about/guidelines.xhtml

APPENDIX B: INTERVIEW TOPIC GUIDE

Background and Previous Experience

1. To start the interview, could you tell me when you were diagnosed with asthma?

2. What type of medications are you currently prescribed for your asthma?

    a. How has your experience been with these medications? (Prompting to see if there are concerns, side effects etc.)

3. A lot of people with asthma have questions or concerns about their inhalers or tablets. What do you do when you have any questions or concerns specifically about your asthma medication?

    a. Prompts: do you do research online? Do you talk to friends and family? Do you go talk to a healthcare professional?
    b. Could you tell me a bit about why you prefer (insert route, e.g. researching online, talking to the GP, talking to family etc.) when it comes to your medication?

4. Have you ever talked to a pharmacist about your asthma medication?

    a. If **YES**: Could you walk me through what happened during that interaction?
        i. Where did it take place? (community pharmacist, hospital pharmacist etc.)
        ii. Prompts: what type of information did they give you? Were they able to answer your questions? Did you feel comfortable talking to them about your asthma medication? Did you feel like it was helpful for you?
        iii. Would you consider the pharmacist as a regular part of your asthma care team? Why or why not?

    b. If **NO**: Could you imagine a pharmacist as part of your regular asthma care team?

5. I'd now like to talk specifically about your preventer inhaler (brown inhaler, steroid inhaler). They're normally prescribed for daily or twice daily use. A lot of asthma patients we've talked to often don't take the inhaler as regularly as prescribed, is this something you've experienced as well?
    a. Could you tell me a little bit about why?

Pharmacist in general practice

Great, that's the first part of our interview. Now we're going to continue on to the part where I'd like to get your thoughts on pharmacist-led consultations in general practice. I've e-mailed you a description of the service already, but would you like to review it to refresh your memory?

For peer review only - http://bmjopen.bmj.com/site/about/guidelines.xhtml

Do you have any questions or anything you would like me to clarify before we move on to the questions?

6. What are your initial thoughts on this pharmacist medication support service?
    a. Explore which aspects of the service are most salient to the participant, use prompts below when needed.

- In your opinion, would this type of service be a useful addition to the care you get from your GP/nurse/asthma specialist?
    - Is this a type of service you could see yourself using? Why or why not?
    - Do you think this service is any different from the asthma care you already receive?
- Do you think this type of service would help you take your preventer inhaler more regularly?
- How do you feel about a pharmacist taking on this type of clinical role?
    - Do you think pharmacists have enough training for this type of work?
        - What type of knowledge would you expect the pharmacist to have?
    - How do pharmacists compare to your other healthcare professionals (e.g. GP or nurse)?
    - Do you think this service is any different from seeing a community pharmacist? (Are they familiar with the NMS and MUR?)
    - Would you feel comfortable talking to a pharmacist about your asthma and your medication?
    - Do you feel like you can trust a pharmacist in this type of role?
- How do you feel about having a pharmacist based in a GP surgery?
    - How would you feel about this pharmacist having access to your medical records?
- What do you think about seeing a pharmacist on an appointment basis?
- When you think about (concerns/side effects discussed for question 2a), do you think those types of questions or concerns could be addressed by a pharmacist?
    - What would be the ideal way to support you with those questions or concerns?
- Do you think this type of service is convenient for people with asthma?

For peer review only

Standards for Reporting Qualitative Research (SRQR)*

http://www.equator-network.org/reporting-guidelines/srqr/

Title and abstract

	Page no.
**Title** - Concise description of the nature and topic of the study Identifying the study as qualitative or indicating the approach (e.g., ethnography, grounded theory) or data collection methods (e.g., interview, focus group) is recommended	0
**Abstract** - Summary of key elements of the study using the abstract format of the intended publication; typically includes background, purpose, methods, results, and conclusions	1

Introduction

**Problem formulation** - Description and significance of the problem/phenomenon studied; review of relevant theory and empirical work; problem statement	2 - 3
**Purpose or research question** - Purpose of the study and specific objectives or questions	3

Methods

**Qualitative approach and research paradigm** - Qualitative approach (e.g., ethnography, grounded theory, case study, phenomenology, narrative research) and guiding theory if appropriate; identifying the research paradigm (e.g., postpositivist, constructivist/ interpretivist) is also recommended; rationale**	3
**Researcher characteristics and reflexivity** - Researchers' characteristics that may influence the research, including personal attributes, qualifications/experience, relationship with participants, assumptions, and/or presuppositions; potential or actual interaction between researchers' characteristics and the research questions, approach, methods, results, and/or transferability	4
**Context** - Setting/site and salient contextual factors; rationale**	4
**Sampling strategy** - How and why research participants, documents, or events were selected; criteria for deciding when no further sampling was necessary (e.g., sampling saturation); rationale**	4-5
**Ethical issues pertaining to human subjects** - Documentation of approval by an appropriate ethics review board and participant consent, or explanation for lack thereof; other confidentiality and data security issues	17
**Data collection methods** - Types of data collected; details of data collection procedures including (as appropriate) start and stop dates of data collection and analysis, iterative process, triangulation of sources/methods, and modification of procedures in response to evolving study findings; rationale**	5

For peer review only - http://bmjopen.bmj.com/site/about/guidelines.xhtml

**Data collection instruments and technologies** - Description of instruments (e.g., interview guides, questionnaires) and devices (e.g., audio recorders) used for data collection; if/how the instrument(s) changed over the course of the study	5
**Units of study** - Number and relevant characteristics of participants, documents, or events included in the study; level of participation (could be reported in results)	6-7
**Data processing** - Methods for processing data prior to and during analysis, including transcription, data entry, data management and security, verification of data integrity, data coding, and anonymization/de-identification of excerpts	5
**Data analysis** - Process by which inferences, themes, etc., were identified and developed, including the researchers involved in data analysis; usually references a specific paradigm or approach; rationale**	5-6
**Techniques to enhance trustworthiness** - Techniques to enhance trustworthiness and credibility of data analysis (e.g., member checking, audit trail, triangulation); rationale**	5-6

Results/findings

**Synthesis and interpretation** - Main findings (e.g., interpretations, inferences, and themes); might include development of a theory or model, or integration with prior research or theory	7-12
**Links to empirical data** - Evidence (e.g., quotes, field notes, text excerpts, photographs) to substantiate analytic findings	7-12

Discussion

**Integration with prior work, implications, transferability, and contribution(s) to the field -** Short summary of main findings; explanation of how findings and conclusions connect to, support, elaborate on, or challenge conclusions of earlier scholarship; discussion of scope of application/generalizability; identification of unique contribution(s) to scholarship in a discipline or field	12 - 17
**Limitations** - Trustworthiness and limitations of findings	14 - 15

Other

**Conflicts of interest** - Potential sources of influence or perceived influence on study conduct and conclusions; how these were managed	17
**Funding** - Sources of funding and other support; role of funders in data collection, interpretation, and reporting	17

*The authors created the SRQR by searching the literature to identify guidelines, reporting standards, and critical appraisal criteria for qualitative research; reviewing the reference lists of retrieved sources; and contacting experts to gain feedback. The SRQR aims to improve the transparency of all aspects of qualitative research by providing clear standards for reporting qualitative research.

For peer review only

**The rationale should briefly discuss the justification for choosing that theory, approach, method, or technique rather than other options available, the assumptions and limitations implicit in those choices, and how those choices influence study conclusions and transferability. As appropriate, the rationale for several items might be discussed together.

Reference:
O'Brien BC, Harris IB, Beckman TJ, Reed DA, Cook DA. **Standards for reporting qualitative research: a synthesis of recommendations.** *Academic Medicine*, Vol. 89, No. 9 / Sept 2014 DOI: 10.1097/ACM.0000000000000388

For peer review only
